# Low-Temperature Synthesis of Magnetic Pyrochlores (R_2_Mn_2_O_7_, R = Y, Ho–Lu) at Ambient Pressure and Potential for High-Entropy Oxide Synthesis

**DOI:** 10.1021/acs.inorgchem.3c00913

**Published:** 2023-06-26

**Authors:** Dovydas Karoblis, Orlando C. Stewart, Priscilla Glaser, Salah Eddin El Jamal, Agne Kizalaite, Tomas Murauskas, Aleksej Zarkov, Aivaras Kareiva, Sarah L. Stoll

**Affiliations:** † Institute of Chemistry, Vilnius University, Naugarduko 24, Vilnius LT-03225, Lithuania; ‡ Department of Chemistry, Georgetown University, 37th and O Streets NW, Washington, D.C. 20057, United States

## Abstract

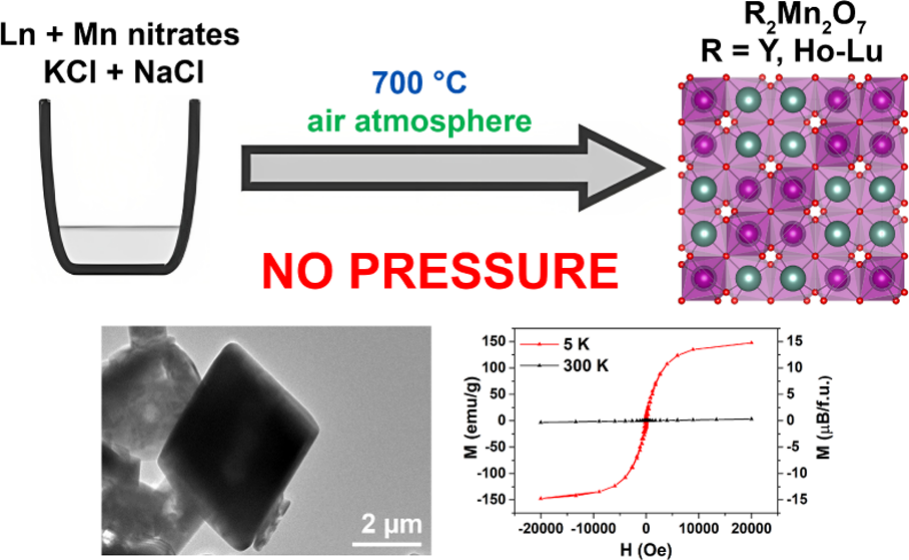

Rare-earth manganese pyrochlores (R_2_Mn_2_O_7_) are frustrated magnetic materials, which previously have only been accessed using expensive high-pressure and high-temperature synthesis. In the present work, we demonstrate a convenient synthetic approach to synthesize R_2_Mn_2_O_7_ pyrochlores at ambient pressure. A series of pyrochlores (R = Y, Ho–Lu) were prepared by a simple and cost-effective molten salt method using NaCl and KCl as the flux. Moreover, phase-selectivity was demonstrated for yttrium manganese oxides (YMnO_3_ and Y_2_Mn_2_O_7_) by a simple variation of synthesis temperature and precursors-to-chlorides ratio. The synthetic procedure does not require high pressures or temperatures nor oxygen flow. All synthesized pyrochlores demonstrated ferromagnetic behavior at low temperature, and the magnetic properties were in good agreement with those of high-pressure-synthesized materials. The versatility of the method was confirmed by the preparation of a mixed-rare earth Y_0.4_Er_0.4_Tm_0.4_Yb_0.4_Lu_0.4_Mn_2_O_7_ solid solution—a compositionally complex high-entropy oxide.

## Abstract 2

Rare-earth manganese pyrochlores (R_2_Mn_2_O_7_) are frustrated magnetic materials, which previously have only been accessed using expensive high-pressure and high-temperature synthesis. In the present work, a series of pyrochlores (R = Y, Ho−Lu) were prepared by a simple and cost-effective molten salt method at ambient pressure. The synthetic procedure does not require high pressures or temperatures nor oxygen flow. The versatility of the method was confirmed by the preparation of a mixed rare-earth Y_0.4_Er_0.4_Tm_0.4_Yb_0.4_Lu_0.4_Mn_2_O_7_ solid solution.

## 1 Introduction

Ternary rare-earth manganese oxides are technologically important materials due to their exceptional properties such as ferroelectricity, magnetism, and reversible magnetocaloric effect.^1−4^ They are widely used in such fields as electronics,^5^ photovoltaics,^6^ and others.^7,8^ The lanthanide–manganese oxides can form various crystalline structures with different manganese oxidation states, *e.g.*, RMnO_3_ polymorphs (Mn^3+^), RMn_2_O_5_ (Mn^3+^, Mn^4+^), and R_2_Mn_2_O_7_ (Mn^4+^); therefore, the selective synthesis and understanding of the formation of particular phases are of high importance and are highlighted in recent reports.^9−11^ Controllable synthesis of metastable materials is of exceptional significance since many solid-state reactions occur at high temperatures (*T* > 1000 °C) to overcome diffusion barriers, resulting in the formation of the most thermodynamically stable phase. Lowering the reaction temperature in solid-state synthesis allows for control over the reaction kinetics to form particular phases. The opportunity to selectively prepare metastable compounds in high purity is critically important for any property-dependent application.^12^

One of the most interesting metastable rare-earth manganese oxides is R_2_Mn_2_O_7_, which exhibits a pyrochlore structure. These materials attracted much attention due to their geometrically frustrated spin structure, which results in interesting physics and broad prospects for the investigation of physical phenomena. Pyrochlore oxides have the chemical composition A_2_B_2_O_7_ and crystallize in the cubic, face-centered crystal structure with the space group *Fd*3̅*m* (#227). Each of the A and B atoms forms an infinite three-dimensional interpenetrating network of corner-shared tetrahedra.^13^ If either the A or B atom is magnetic, then there is a very high degree of frustration when the nearest-neighbor interactions are antiferromagnetic.^14^

The perovskite manganese oxides (R^3+^Mn^3+^O_3_) are relatively easily prepared under ambient oxygen, and phase-pure materials can be obtained by soft chemical approaches such as sol–gel^15^ or hydrothermal^16^ methods rather than through conventional solid-state reactions. By contrast, the synthesis of the Mn-based pyrochlores is considered as a significant challenge. Due to the thermodynamic instability at ambient pressure at any temperature, this group of materials is prepared using high-pressure methods. Empirically, for the R_2_M_2_O_7_ phase, the ratio of the ionic radii between R^3+^ and M^4+^ limits the stability of the pyrochlore structure, and for the small M = Mn^4+^, even the smallest lanthanides (Dy–Lu) require high pressures.^13^ Recently, Pomjakushina *et al.*(17) demonstrated that Tm_2_Mn_2_O_7_ can be prepared through the conversion of TmMnO_3_ at a relatively low oxygen pressure (1100 °C, 1300 bar) to stabilize Mn(IV). This report provides a simplified pyrochlore synthesis; however, the approach was demonstrated for only one member of the series. Alternatively, Todd and Neilson^18^ employed a metathesis reaction for the phase-selective synthesis of yttrium manganese oxides including orthorhombic o-YMnO_3_, hexagonal h-YMnO_3_, and Y_2_Mn_2_O_7_. The selectivity was achieved by varying the synthetic conditions and the alkali metal carbonate precursors. Their work showed that Y_2_Mn_2_O_7_ can be prepared without high pressures although still demanding an oxygen flow. In addition, the less stable o-YMnO_3_ exhibited a contraction either due to cation vacancies or excess oxygen or due to lithium doping from the precursor. A wider range of lanthanides was not demonstrated, another limiting feature.

In the present work, we report a low-temperature, simple, time- and cost-effective approach for the selective preparation of R_2_Mn_2_O_7_ pyrochlores by the molten salt method for R = Y and Ho–Lu. Although the molten salt route is well-known for the synthesis of complex oxides,^19^ the successful synthesis of lanthanide manganese pyrochlores has not been achieved. One of the distinct advantages of our suggested approach is that it does not require high pressure or oxygen flow or other additional oxidants. Moreover, for R = Y, we demonstrate the phase-selective synthesis of o-YMnO_3_, h-YMnO_3_, and Y_2_Mn_2_O_7_. The purity and phase control are confirmed by X-ray powder diffraction and vibrational spectroscopy. The magnetic ordering and oxidation state information was determined using magnetic susceptibility, and we used a combination of scanning electron microscopy (SEM) as well as transmission electron microscopy (TEM) to study the morphology of these materials. Finally, we also show that high-entropy A-site solid solutions can be easily prepared by this method, which requires only a laboratory furnace, sodium and potassium chlorides, and the nitrates of R and Mn ions.

## 2 Experimental Section

### 2.1 Materials

For the preparation of YMnO_3_ polymorphs and R_2_Mn_2_O_7_ pyrochlores, yttrium(III) nitrate hexahydrate [Y(NO_3_)_3_·6H_2_O, Sigma-Aldrich, 99.9%], holmium(III) oxide (Ho_2_O_3_, Sigma-Aldrich, 99.9%), erbium(III) nitrate pentahydrate [Er(NO_3_)_3_·5H_2_O, Sigma-Aldrich, 99.9%], thulium(III) nitrate pentahydrate [Tm(NO_3_)_3_·5H_2_O, Sigma-Aldrich, 99.9%], ytterbium(III) nitrate pentahydrate [Yb(NO_3_)_3_·5H_2_O, Sigma-Aldrich, 99.9%], lutetium(III) oxide (Lu_2_O_3_, Sigma-Aldrich, 99.9%), and manganese(II) nitrate tetrahydrate [Mn(NO_3_)_2_·4H_2_O, Alfa Aesar, 99.9%] were used as starting materials. Sodium chloride (NaCl, Carl Roth, ≥99.5%) and potassium chloride (KCl, Carl Roth, ≥99.5%) were used as a synthesis medium.

### 2.2 Synthesis

For the synthesis of R_2_Mn_2_O_7_ pyrochlores, lanthanide (including yttrium) and manganese nitrates were taken with a molar ratio of 1:1 and thoroughly mixed with sodium and potassium chlorides in an agate mortar; the molar ratio between starting metal nitrates and chlorides was 1:2 and the molar ratio between NaCl and KCl was 1:1. The obtained mixture was transferred into the corundum crucible and annealed at 700 °C for 10 h in air with a heating rate of 5 °C/min. After the annealing procedure, the furnace was cooled down naturally. When lanthanide oxide was used as the starting material (for Ho and Lu), it was converted to nitrate by dissolving it in concentrated nitric acid. After the complete dissolution of the oxide, manganese nitrate was added and the resulting solution was evaporated to dryness. The obtained mixture of nitrates was then dried in an oven, ground in an agate mortar with NaCl and KCl, and annealed at identical conditions.

For the preparation of YMnO_3_ polymorphs, different annealing temperatures and ratios between metal precursors and chlorides were adjusted. The o-YMnO_3_ was synthesized at 800 °C, whereas h-YMnO_3_ was obtained at 1100 °C. In both cases, the molar ratio between the starting metal nitrates and chlorides was 1:20. For the synthesis of both materials, the reaction mixture was annealed for 10 h in air with a heating rate of 5 °C/min. After annealing, the furnace was cooled down naturally.

The obtained products were washed with hot distilled water to dissolve alkali metal chlorides. The resulting powders were filtered and the presence of residual chloride ions was determined using a silver nitrate solution. All powders were then dried in an oven at 80 °C and ground in an agate mortar.

### 2.3 Characterization

Powder X-ray diffraction (XRD) analysis was performed using a Rigaku MiniFlex II diffractometer (Cu Kα, λ = 1.5419 Å) working in Bragg–Brentano (θ/2θ) geometry. The data were collected within the 10–80° 2θ range with a step of 0.02° and scanning speed of 1°/min. The FullProf Suite was used for structural refinement (FullProf Suite software version September 2020). Elemental composition and chemical states of the elements were investigated by X-ray photoelectron spectroscopy (XPS) using a Kratos Axis Supra spectrometer with monochromatic Al Kα (25 mA, 15 kV). The instrument was calibrated using metallic gold and copper samples. The measurements were carried out with a charge neutralization, while the energy scale was charge-corrected to the main line of carbon (C 1s) at 284.8 eV. Fourier transform infrared (FTIR) spectra were obtained in the range of 4000–400 cm^–1^ with a Bruker ALPHA-FTIR spectrometer with 4 cm^–1^ resolution. Raman spectra were recorded using a combined Raman and scanning near-field optical microscope WiTec Alpha 300 R equipped with a 532 nm excitation laser source. The morphology of the synthesized powders was analyzed by SEM using a Hitachi SU-70 microscope. HRTEM and energy-dispersive spectrometry (EDS) were performed on a JEOL JEM 2100F FEG TEM/STEM instrument operated at 200 kV. Magnetic susceptibility data were collected using a Quantum Design MPMS3 SQUID magnetometer. Data were collected using a temperature sweeping mode from 5 to 300 K at 50 Oe under both zero-field cooled (ZFC) and field-cooled warming (FCW) conditions. FCW data were collected by cooling the samples to 5 K in a field of 50 Oe and measuring the moment as the sample was heated to 300 K. The Curie–Weiss analysis was done on field-cooled (FC) data at 5000 Oe. Magnetic hysteresis data were collected at 5 K from −20 000 to 20 000 Oe. Data were collected in VSM mode with 5 mm scan lengths and 2 s averaging or in DC scan mode with 30 mm scan lengths and 5 s averaging. All data were corrected for diamagnetic contributions using Pascal’s constants^20^ and for sample shape and radial offset effects using the MPMS3 Sample Geometry Simulator.^21^ Curie–Weiss analyses were performed by plotting 1/χ *vs T* data collected at 5000 Oe and fitting the linear portion from 100 to 300 K.

## 3 Results and Discussion

**Figure 1:**
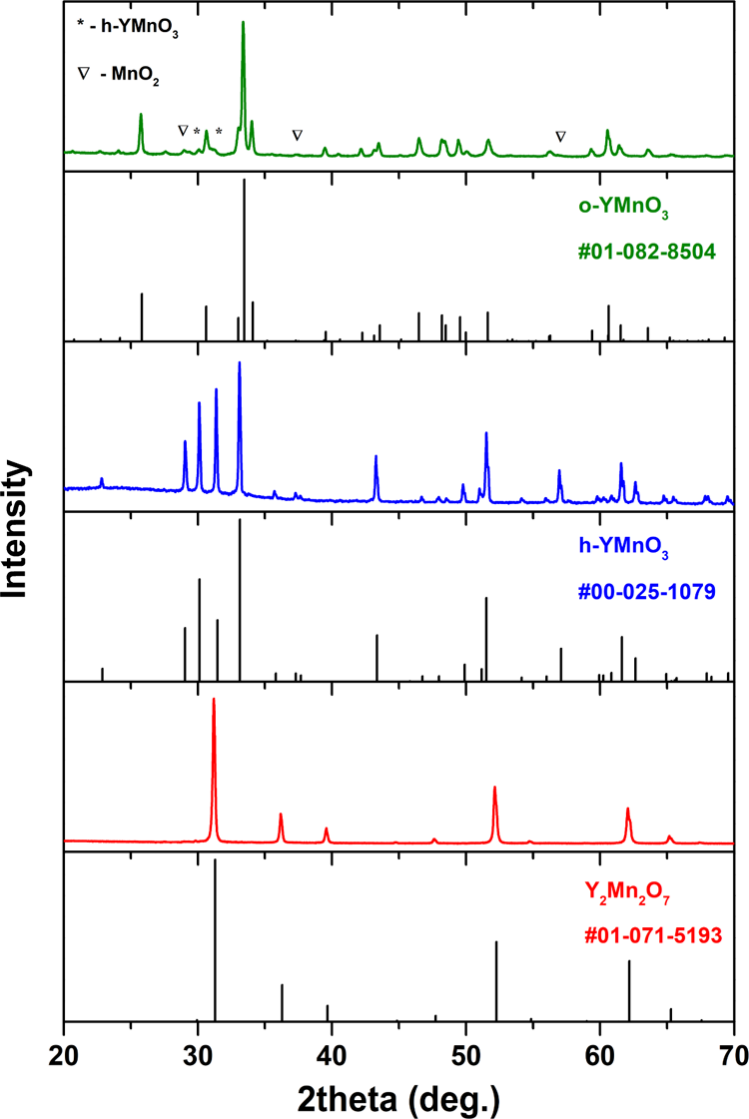
XRD patterns for different synthetic conditions: 800 °C, o-YMnO_3_ (green); 1100 °C, h-YMnO_3_ (blue); 700 °C and high concentration, Y_2_Mn_2_O_7_ (red).

The yttrium manganese oxides were selected as a model system to demonstrate phase control because of the sensitivity to the reaction pathway.^18^ It is known that larger lanthanides tend to form orthorhombic perovskites (o-RMnO_3_) whereas smaller ones preferably crystallize in a hexagonal structure (h-RMnO_3_).^22^ YMnO_3_ can possess both these structures, and although o-YMnO_3_ is assumed to be metastable, it can be obtained by either high-pressure synthesis or soft chemistry procedures.^23^ Upon annealing under ambient pressure, o-YMnO_3_ transforms to h-YMnO_3_, and the phase transition occurs at around 1123 K; therefore, the most straightforward way to control the formation of either polymorph is to change the synthesis temperature. Figure 1 depicts the XRD patterns of yttrium manganese oxides synthesized under different conditions. Clearly, at 800 °C, o-YMnO_3_ was obtained; the most intense diffraction peaks are in a good agreement with the standard XRD data (PDF no. 01-082-8504). However, some minor phases such as h-YMnO_3_ and MnO_2_ were also observed. When the synthesis temperature was increased to 1100 °C, h-YMnO_3_ was obtained. The positions of diffraction peaks match very well with the PDF no. 00-025-1079 data. Thus, both YMnO_3_ polymorphs with Mn ions in oxidation state 3+ can be easily obtained by changing the synthesis temperature. Alternatively, when the concentration of nitrates precursors was increased (nitrates-to-chlorides molar ratio 1:2), the product was the single-phase Y_2_Mn_2_O_7_ pyrochlore. All diffraction peaks correspond to cubic Y_2_Mn_2_O_7_ with the space group *Fd*3̅*m* (no. 227); the intensity and positions of the peaks match very well with the standard data (PDF no. 01-071-5193). The formation of this material suggests that Mn ions are oxidized from the precursor 2+ to the 4+ state in the product. Besides the use of high pressure, previous successful syntheses of cubic R_2_Mn_2_O_7_ are associated with using strong oxidizing agents such as KIO_4_,^24^ NaOH and NaClO_3_^25^ or O_2_ pressure^17,26^ even when Mn(IV) oxide was used as a starting material.^24−26^ The softest reported approach demonstrated by Todd and Neilson^18^ also employed oxygen flow for the synthesis of Y_2_Mn_2_O_7_. Here, the synthesis is performed in air under atmospheric pressure. We hypothesize that the *in situ* oxidation of Mn ions to the 4+ state could be related to the nature of metal precursors and the mechanism of decomposition of nitrates, which results in the oxidation of Mn^2+^ ions.

To confirm the role of the nitrates, we performed the synthesis under identical conditions using metal chlorides (YCl_3_ and MnCl_2_) and oxides (Y_2_O_3_ and MnO_2_) as starting materials. These reagents did not lead to the formation of the pyrochlore structure but rather resulted in the crystallization of o-YMnO_3_ (Figure S1). When only manganese nitrate was treated in molten salts (without yttrium nitrate), the resulting product was a mixture of MnO_2_ and Mn_2_O_3_. The mixed oxidation states of Mn suggest that the amount of nitrates was insufficient for the complete oxidation of Mn^2+^ ions. For targeting the pyrochlore, the nitrates-to-alkali chlorides molar ratio was also important. Single-phase Y_2_Mn_2_O_7_ was obtained when the molar ratio of Y and Mn nitrates to NaCl and KCl was 1:2. Lowering this ratio (less nitrates) led to the dilution of nitrates, and the percentage of pyrochlore gradually decreased with decreasing concentration (Figure S2). When the ratio dropped below 1:20, the major crystalline phase was o-YMnO_3_.

**Figure 2:**
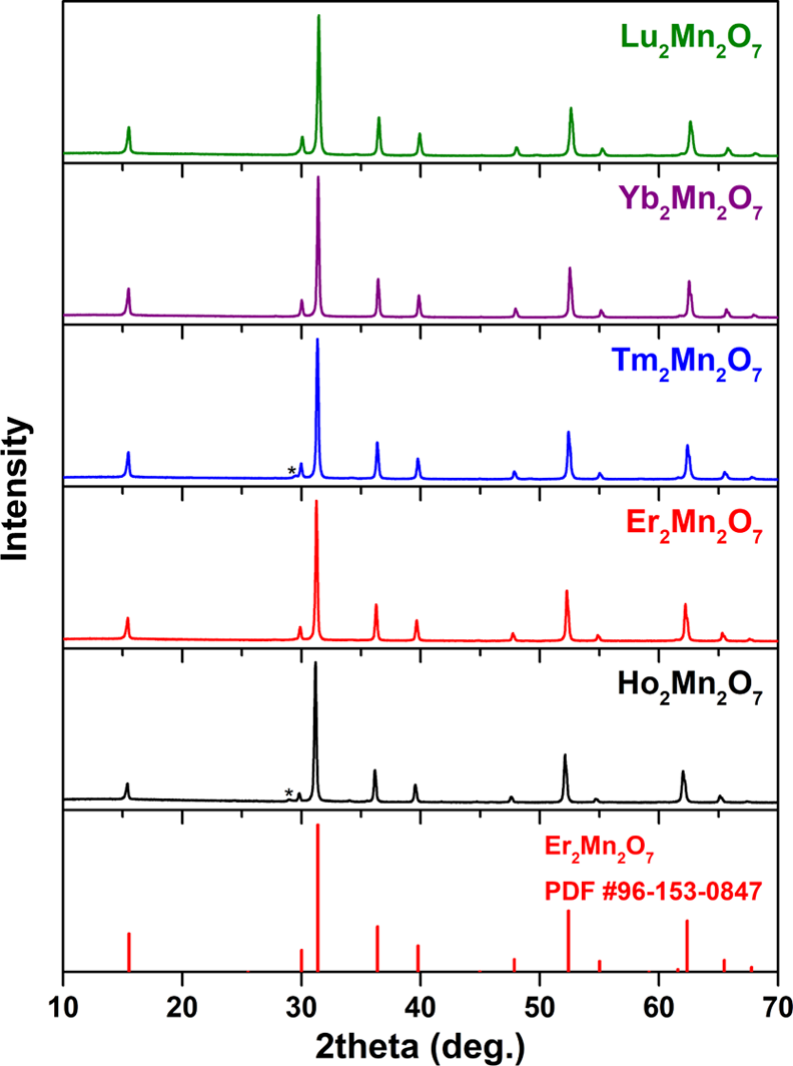
XRD patterns of R_2_Mn_2_O_7_ (R = Ho–Lu). The asterisks denote the relevant Ln_2_O_3_ phase.

The optimized pyrochlore synthesis for yttrium manganese oxides was tested with other rare-earth elements to confirm the generality and suitability of the synthetic approach. Figure 2 shows the XRD patterns of R_2_Mn_2_O_7_ (R from Ho to Lu). In some patterns, negligible amounts of the lanthanide sesquioxide phase can be detected; however, it is evident that the major pyrochlore phase was obtained in all cases, with no evidence of the RMnO_3_ phases. As found in the solid state, the synthesis of pyrochlores with larger lanthanides was not successful. To the best of our knowledge, Dy is the largest lanthanide ion forming the R_2_Mn_2_O_7_ structure,^25^ however, this method did not produce phase-pure Dy_2_Mn_2_O_7_. Although the pyrochlore phase was evident, significant amounts of DyMnO_3_ and DyMn_2_O_5_ were also present (Figure S3). Rietveld refinement was performed for all synthesized pyrochlores (Figures S4–S9), and the calculated cell parameters are summarized in Table 1. The lattice constants for the corresponding materials synthesized using high-pressures reported by Subramanian *et al.*(25) are provided for comparison. As expected, the cell parameters in the lanthanides’ row gradually decrease from Ho to Lu, which is in good agreement with their ionic radii,^27^ and there is no evidence for non-stoichiometry. The unit cell of pyrochlores is sensitive to composition, and the consistency in the cell constant suggests that there is neither oxygen deficiency (which would increase the cell) nor cation vacancies (which would decrease the cell).^13^

**Table 1:** Cell Parameters of Synthesized R_2_Mn_2_O_7_

sample	*a* (Å)	*V* (Å^3^)	*a* (Å), ref (25)
Y_2_Mn_2_O_7_	9.8962(7)	969.20(2)	9.901
Ho_2_Mn_2_O_7_	9.899(86)	970.2(57)	9.905
Er_2_Mn_2_O_7_	9.8704(7)	961.64(1)	9.869
Tm_2_Mn_2_O_7_	9.8467(3)	954.72(1)	9.847
Yb_2_Mn_2_O_7_	9.8251(4)	948.4(53)	9.830
Lu_2_Mn_2_O_7_	9.803(48)	942.1(96)	9.815

**Figure 3:**
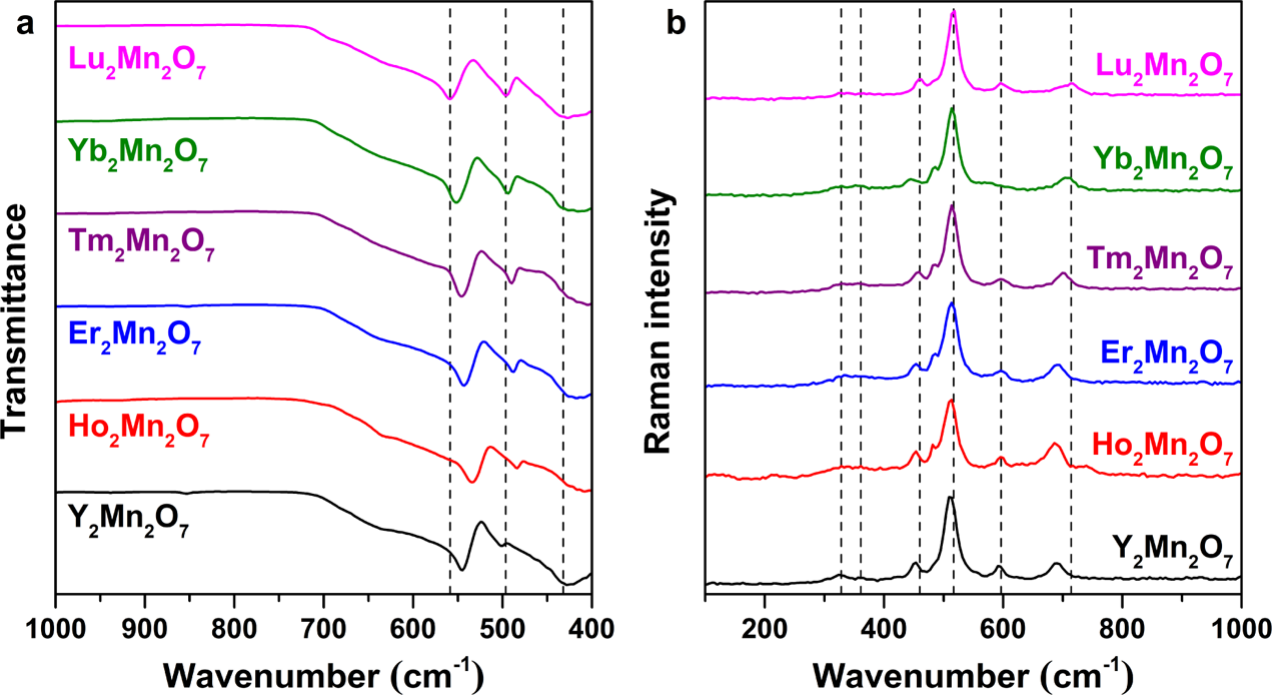
FTIR (a) and Raman (b) spectra of R_2_Mn_2_O_7_.

Vibrational spectroscopy provides information regarding the distribution of cations located in different sites, distortion of local symmetry, or any short-range order. Out of the 26 normal modes predicted by the group-theoretical analysis for pyrochlore-type materials,^28^ only 7 are IR-active (namely, 7F_1u_) and 6 are Raman-active (A_1g_, E_g_, and 4F_2g_). According to the previous study by Brown *et al.*,^29^ 3 active IR modes can be found in the 460–570 cm^–1^ range, while the remaining bands are located in the far-infrared region. In the R_2_Mn_2_O_7_ case (Figure 3a), we observed 3 absorption bands centered in the range from 410 to 560 cm^–1^ (Table 2). The intense band centered at 560 cm^–1^ (for Lu_2_Mn_2_O_7_) is associated with the Mn–O stretching vibration (F_1u_^1^), while two others at 497 and 428 cm^–1^ are attributed to R–O′ (F_1u_^2^) and R–O (F_1u_^3^) stretching vibrations, respectively. Here, having the complete series of R_2_Mn_2_O_7_ provides an opportunity to observe trends as a function of R radii. First, an increase in the lanthanide ionic radius leads to a shift to lower wavenumbers for all absorption bands. Moreover, the intensity of the band located in the ∼483–497 cm^–1^ range decreases as R increases from Lu to Ho. Although the ionic radius of Y^3+^ is the largest in the investigated series, Y_2_Mn_2_O_7_ does not follow this trend. According to the previous work by Subramanian *et al.*,^30^ the location of the band maxima can be influenced not only by the ionic radius of the A-site cation but also by the mass of this ion. Since Y is about two times lighter than the closest in ionic radius Ho, this could explain the difference in peak positions for Y_2_Mn_2_O_7_.

**Table 2:** Band Wavenumbers (cm^–1^) and Assignments of the FTIR and Raman Spectra of R_2_Mn_2_O_7_

	Y_2_Mn_2_O_7_	Ho_2_Mn_2_O_7_	Er_2_Mn_2_O_7_	Tm_2_Mn_2_O_7_	Yb_2_Mn_2_O_7_	Lu_2_Mn_2_O_7_
FTIR						
F_1u_^1^	544	535	542	546	552	560
F_1u_^2^	503	483	488	490	494	497
F_1u_^3^	430	410	414	416	422	428
Raman						
A_1g_	510	512	513	514	514	518
E_g_ + F_2g_^4^	325	328	334	328	329	326
F_2g_^1^	591	596	596	597	581	598
F_2g_^2^	452	453	455	458	444	459
F_2g_^3^	356	361	365	356	351	365

We also compared the Raman spectra of R_2_Mn_2_O_7_, as seen in Figure 3b. Neither R^3+^ or Mn^4+^ ions contribute to the allowed fundamental transitions, which means that a change in the A-site cations (R) leads to small variations in band positions and intensity in Raman spectra. According to previous detailed spectroscopic analysis of some pyrochlores, R_2_Mn_2_O_7_ (R = Y, Dy, Er, Yb, In, Tl), by Brown *et al.*,^29,31^ six Raman-active bands were observed in the 300–600 cm^–1^ range, which appear to overlap in our study. The most intense peak (A_1g_), which arises due to the bending of Mn–O_6_ octahedra, was observed at 510–518 cm^–1^ for all compounds (Table 2). Although XRD analysis revealed a small amount of the lanthanide sesquioxide phase, there were no signals corresponding to this neighboring phase in the Raman spectra, and the most intense band for R_2_O_3_ should occur at ∼320–420 cm^–1^ depending on R.^32^ Additionally, for some R_2_Mn_2_O_7_ (R = Ho, Er, Tm, Yb) a low-intensity band at the 481–485 cm^–1^ region was observed, which was not reported in previous studies as characteristic of the pyrochlore phase. The origin of this band is unclear; however, it is not associated with R_2_O_3_ nor manganese oxide phases.^33^

**Figure 4:**
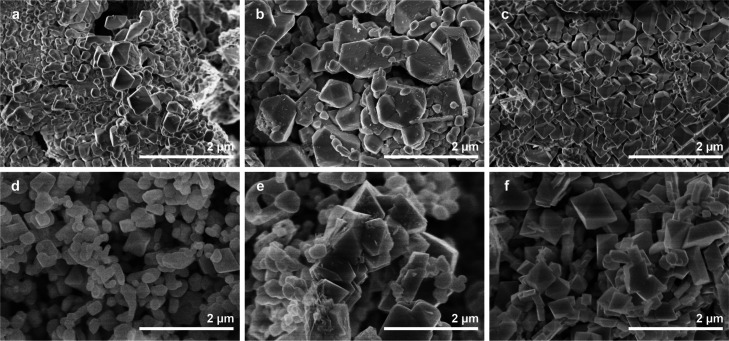
SEM images of Y_2_Mn_2_O_7_ (a); Ho_2_Mn_2_O_7_ (b); Er_2_Mn_2_O_7_ (c); Tm_2_Mn_2_O_7_ (d); Yb_2_Mn_2_O_7_ (e); and Lu_2_Mn_2_O_7_ (f).

The morphology of the materials was characterized using SEM, which is shown in Figure 4. Cubic crystals can grow as cubes when the [100] face grows more rapidly or as octahedra when the fastest growing face is the [111] face.^34^ The primary shape observed was octahedral crystals although samples did have polyhedral particles with neighboring particles of an undefined shape.

Interestingly, across the series of lanthanides, the pyrochlores with a smaller R tended to form more well-faceted particles of a defined shape, whereas pyrochlores with a larger R contained more particles of an irregular shape. The size of particles varied from approximately micron- to nanoscale. There has been little comment about the morphology of R_2_Mn_2_O_7_ pyrochlores synthesized under high pressures; however, Subramanian *et al.*(25) mentioned crystals with an octahedral shape.

**Figure 5:**
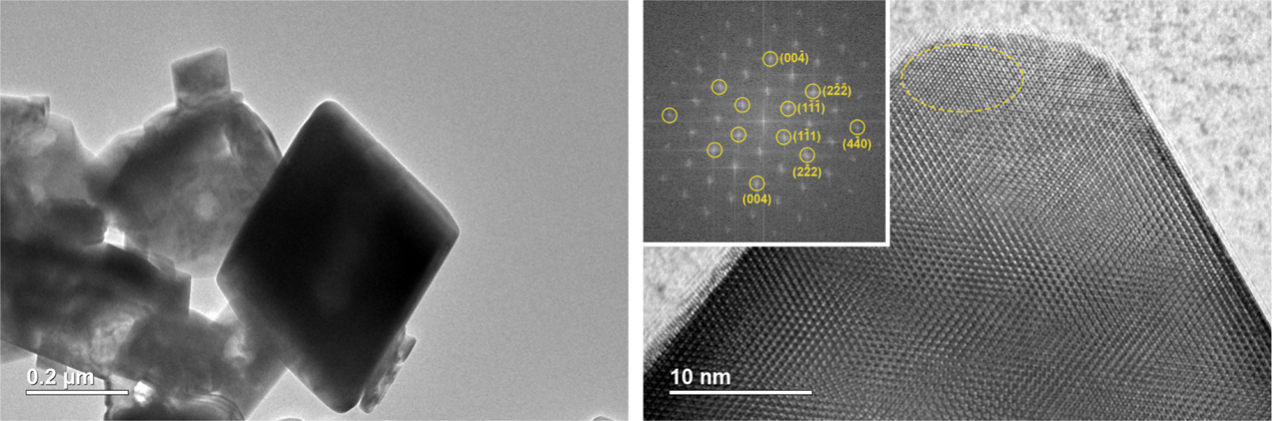
TEM images of Lu_2_Mn_2_O_7_.

The TEM studies are consistent with those of SEM. For example, a representative TEM of Lu_2_Mn_2_O_7_ shows a particle of octahedral shape in Figure 5. In addition, the HRTEM image reveals that the synthesized material contains single crystals. Both fast Fourier transform (FFT) and inverse FFT analyses were performed to evaluate the interplanar spacing. The *d*-spacing was determined to be ∼0.5 nm, which can be assigned to the (111) plane of Lu_2_Mn_2_O_7_. The obtained FFT image also displays evidence of double diffraction, which is common for materials containing heavy elements and thick samples.

**Figure 6:**
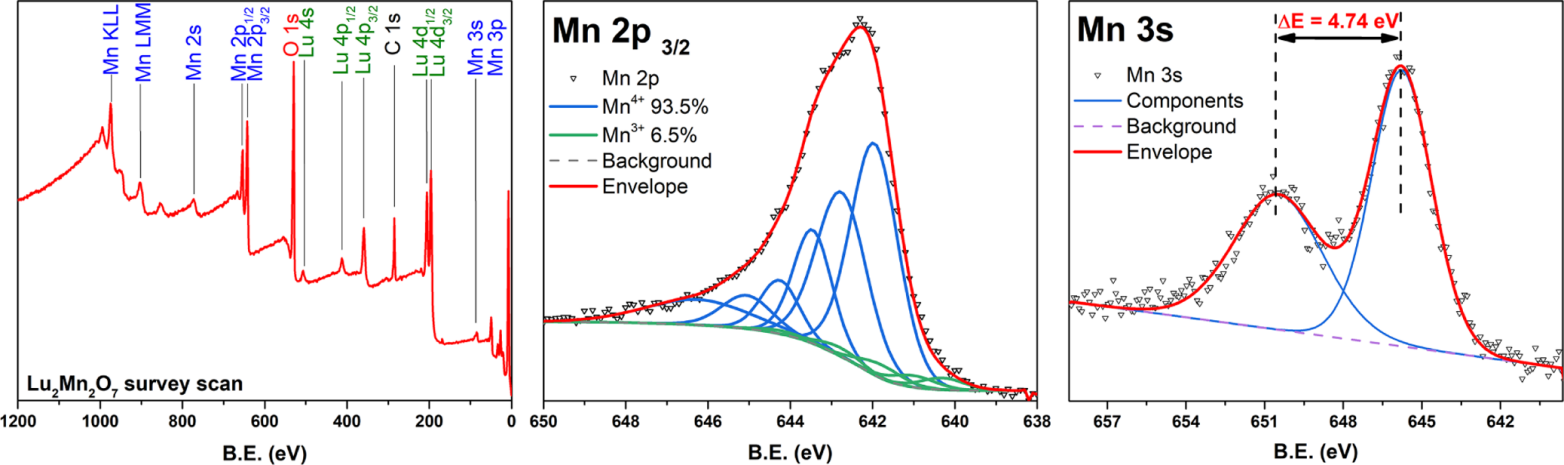
XPS spectra of Lu_2_Mn_2_O_7_.

To confirm the purity of the pyrochlores, which contain only Mn(IV), XPS spectra were recorded for the series (Figure 6). An adventitious carbon signal was the only additional signal unrelated to the compound-constituting elements such as R, Mn, and O. Importantly, neither sodium or potassium was detected, consistent with the cell constants, confirming that there was no adventitious alkali metal doping. To determine the oxidation state of manganese, high-resolution Mn 2p_3/2_ and Mn 3s were recorded. As found for many other transition metals, the spectra of Mn 2+, 3+, and 4+ demonstrate not only spin–orbit splitting, resulting in Mn 2p_3/2_ and Mn 2p_1/2_ peaks, but also multiplet splitting. Each manganese oxidation state of 2+, 3+, and 4+ in the Mn 2p_3/2_ consists of multiple basic components, where Mn 2+ also exhibits a satellite peak of higher binding energy.^35^ All of the states are generally characterized by very similar binding energies, making Mn analysis quite complicated. Thus, to correctly evaluate instrumental errors and obtain typical full width at half-maximum (FWHM) values, an additional MnO_2_ standard sample was measured. The lack of satellite features in the Mn 2p_3/2_ spectra of all the samples suggests that the presence of the Mn 2+ state is highly unlikely. Thus, MnO_2_ and the rest of the spectra were fitted using the Mn 3+/4+ model similar to MnO_2_ due to the dominant amount of Mn 4+.^36^ The XPS spectra of Lu_2_Mn_2_O_7_ are given in Figure 6 as representative. A small percentage of Mn 3+ (5–10%) was detected in the samples and the standard, which is most likely related with manganite formation on the surface or X-ray-induced reduction. These results were further supported by the peak orbital splitting measurements in Mn 3s high-resolution spectra. Mn 3s spectra splitting results from spin coupling between electrons in the 3s and 3d orbitals. The binding energy difference (Δ*E*) between two 3s peaks is typically related to Mn-oxidation state and could, to some degree, be an indicator of the manganese oxidation state in oxide compounds.^37^ The Mn 3s energy difference amounts to ∼4.7 eV in MnO_2_ samples and increases drastically as the Mn is reduced to 3+ or 2+ to Δ*E* ∼ 5.9 eV.^38^ In the R_2_Mn_2_O_7_ sample and standard MnO_2_, the measured Δ*E* ≅ 4.7 eV. All of the Mn spectra indicate that the major amount of manganese is in the tetravalent state (Mn^4+^), which also agrees well with the results of structural analysis. The results of XPS analysis for the rest of the synthesized pyrochlores (Figure S10) were comparable with those given for Lu_2_Mn_2_O_7_.

**Figure 7:**
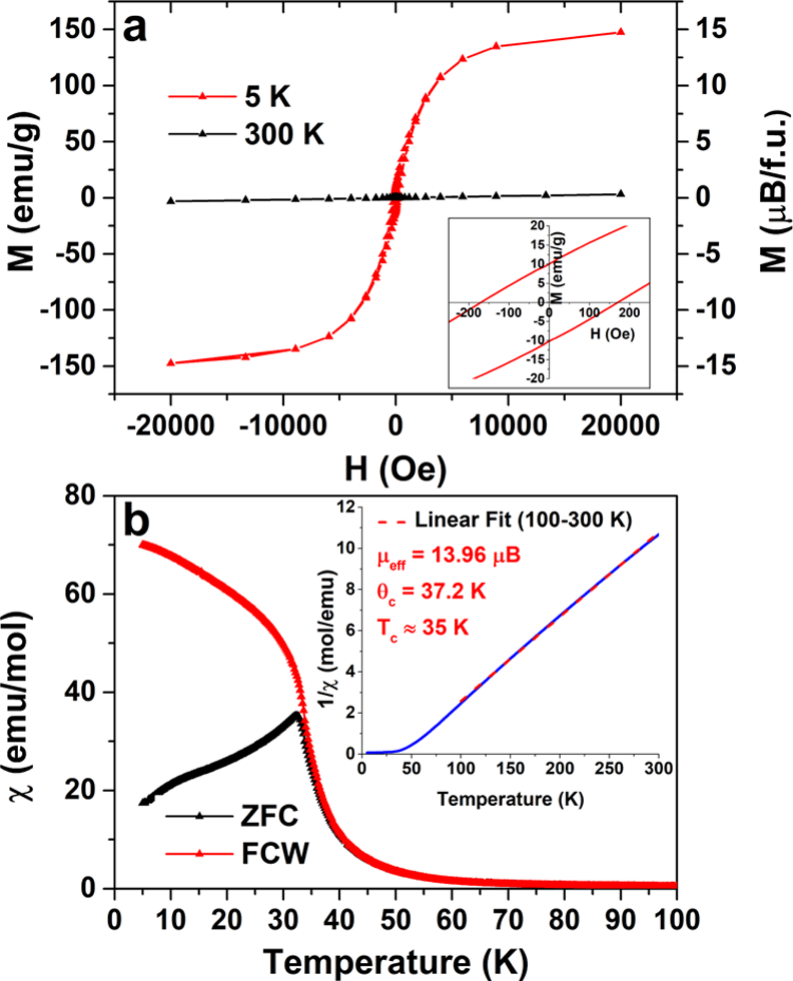
*M vs H* curves of Er_2_Mn_2_O_7_ at 5 and 300 K. Inset: zoomed-in curve at 5 K (a); magnetic susceptibility of Er_2_Mn_2_O_7_*vs T* at 50 Oe for ZFC and FC data. Inset: inverse magnetic susceptibility of Er_2_Mn_2_O_7_*vs T* at 5000 Oe with Curie–Weiss analysis and linear fit from 100 to 300 K (b).

The magnetic properties of the prepared pyrochlores were measured and compared with analogues synthesized by high pressures. The R_2_Mn_2_O_7_ materials exhibit a cooperative two-sublattice ferromagnetism that is second-order, which can lead to a strong magneto-caloric effect.^4^ Since Lu^3+^ and Y^3+^ are nonmagnetic ions with μ_eff_ = 0, the magnetic susceptibility of Lu_2_Mn_2_O_7_ and Y_2_Mn_2_O_7_ are only due to Mn magnetic moments, while the magnetic properties of other pyrochlores are influenced by both cations. Based on neutron diffraction studies, the Mn(IV) sublattice ferromagnetically orders, with a moment close to the full spin-only value (3.0 μB per Mn) and a reduced moment for the rare earth.^13^ As a result, the μ_eff_ (from Curie–Weiss analysis) and *M*_sat_ (*M*-*vs*-*H*) both tend to be lower than predicted and ascribed to a reduced lanthanide moment. The representative magnetic data obtained for Er_2_Mn_2_O_7_ is given in Figure 7, and the corresponding magnetic data for other pyrochlores can be found in Figures S11–S25. The magnetic measurements are consistent with other data reported here that support the fact that the R_2_Mn_2_O_7_ materials are stoichiometric with no alkali metal doping. The magnetization *versus* field curves (at 5 K) confirm that the series of R_2_Mn_2_O_7_ are ferromagnetic, and these data were used to determine the saturation magnetization (5.2–14.7 μ_B_/f.u.) and coercivities (14–1050 Oe) (Table 3). The saturation magnetization values obtained in our work are slightly lower than the values reported for R_2_Mn_2_O_7_ (R = Ho–Yb) in previous studies,^4,39,40^ which may be attributed to the lack of full saturation at our maximum applied magnetic field (2 T).

**Table 3:** Magnetic Quantities for Rare-Earth Pyrochlores in This Study^a^

pyrochlore	*T*_c_ (K)	*T*_c_ (ref (42))	μ_eff_ (μ_B_)	μ_eff_ (ref (42))	θ_CW_ (K)	*M*_sat_ (emu/g)	*M*_sat_ (μ_B_/f.u.)	*H*_c_ (Oe)
Y_2_Mn_2_O_7_	∼20	20	5.66	5.4	52.2	75	5.3	14
Ho_2_Mn_2_O_7_	∼40	37	15.0	14.4	42.7	135	13.4	1050
Er_2_Mn_2_O_7_	∼35	35	13.9	13.3	37.2	145	14.7	350
Tm_2_Mn_2_O_7_	∼30	30	11.5	10.4	35.9	95	9.5	175
Yb_2_Mn_2_O_7_	∼40	35	7.80	7.6	50.0	85	8.7	65
Lu_2_Mn_2_O_7_	∼20	23	5.52	4.9	59.9	50	5.2	28

a Saturation magnetization (*M*_sat_) and coercivities (*H*_c_) were determined at 5 K. Curie temperatures (*T*_c_), effective moments (μ_eff_), and Curie–Weiss temperatures (θ_CW_) were determined at 5000 Oe.

The ferromagnetic ordering temperature was determined from the maximum in the slope of the χ-*vs*-*T* plots, and the Curie–Weiss law was applied to the paramagnetic region for 1/χ *vs T* data (inset of Figure 7b) to determine Curie–Weiss temperatures θ_CW_ and effective moments μ_eff_, as summarized in Table 3. The *T*_c_ values are close to those reported, and the Weiss constants are positive as expected for ferromagnetic coupling. The level of magnetic frustration is often measured by the frustration index (*f* = |θ_CW_|/*T*_c_).^13^ In agreement with prior reports, R_2_Mn_2_O_7_ with magnetic metals for R have an index in a range close to 1 (here 1.1–1.4), which is normal for a ferromagnet. By contrast, for non-magnetic metals, R = Y and Lu are notably larger (here 2.14–2.6), suggesting a more complex magnetic coupling for Mn.^13^ Consistent with the saturation magnetization measurements, the effective moment (μ_eff_) is slightly smaller than previously reported.^25^ Finally, we include the warming data for both FC and ZFC samples, as shown in Figure 7b. Splitting between the ZFC and FC curves was observed for all pyrochlores in the 20–40 K range. The extent of splitting depends on R and has been attributed to magnetocrystalline anisotropy, magnetic domain formation,^4^ additional R–R or R–Mn interactions,^40,41^ or spin glass behavior.^40^

**Figure 8:**
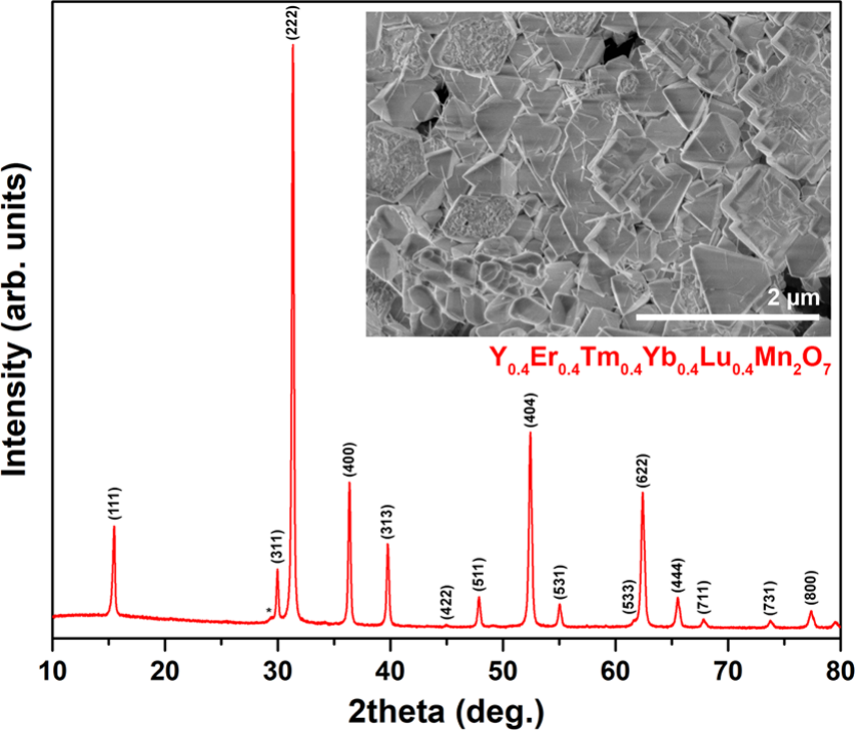
XRD pattern of the Y_0.4_Er_0.4_Tm_0.4_Yb_0.4_Lu_0.4_Mn_2_O_7_ solid solution. Inset: SEM image of the Y_0.4_Er_0.4_Tm_0.4_Yb_0.4_Lu_0.4_Mn_2_O_7_ solid solution. The asterisk denotes the Ln_2_O_3_ phase.

Finally, we were interested in determining whether the magnetic pyrochlores, R_2_Mn_2_O_7_, would allow for chemical disorder, to form a high entropy oxide,^43^ with multiple metals on the R site as demonstrated for other pyrochlores,^44^ for phase stabilization and tuning of magnetic properties.^45^ To our knowledge, A-site solid solutions of R_2_Mn_2_O_7_ were synthesized and investigated only in one work by Imamura *et al.*,^41^ who investigated three binary solid solutions, namely, (Y_1–*x*_Lu_*x*_)_2_Mn_2_O_7_, (Dy_1–*x*_Yb_*x*_)_2_Mn_2_O_7_, and (Dy_1–*x*_Lu_*x*_)_2_Mn_2_O_7_ and demonstrated that the magnetic properties of pyrochlores can be tuned by A-site substitution. Due to recent interest in magnetic high-entropy oxides^44−46^ and to demonstrate the versatility of our synthetic approach, a Y_0.4_Er_0.4_Tm_0.4_Yb_0.4_Lu_0.4_Mn_2_O_7_ solid solution was synthesized. The XRD pattern is shown in Figure 8, and it is seen that all intense diffraction peaks correspond to cubic pyrochlore structure; however, a negligible amount of Ln_2_O_3_ is also observed, like in the case of some single-lanthanide pyrochlores (Figure 2). The diffraction peaks were indexed according to the Er_2_Mn_2_O_7_ standard XRD data (no. 96-153-0847), and the Rietveld refinement data are given in Figure S26. As evidence of the formation of solid solution, instead of a mixture of 5 separate pyrochlores, FWHMs of the most intense [222] peaks were calculated for single-lanthanide pyrochlores and the solid solution. Due to the difference in ionic radius,^27^ there is a systematic peak shift across the row (Figure S27); therefore, a mixture of different pyrochlores is expected to give broad peaks due to the overlapping. The FWHM value for the solid solution was determined to be 0.239, whereas these values for single-lanthanide pyrochlores varied in the range from 0.212 to 0.243°. The cell parameter *a* was calculated as 9.8499 Å and was close to that of Tm_2_Mn_2_O_7_ (see Table 1), which is in the middle of the rare-earths used.

The morphology of the Y_0.4_Er_0.4_Tm_0.4_Yb_0.4_Lu_0.4_Mn_2_O_7_ solid solution was similar to that of the observed for single A-element materials; the SEM image (inset of Figure 8) demonstrates the formation of sub-micrometric particles with a well-defined polyhedral shape. A minor amount of needle-shaped particles is also observed, suggesting the presence of a negligible amount of impurities.

**Figure 9:**
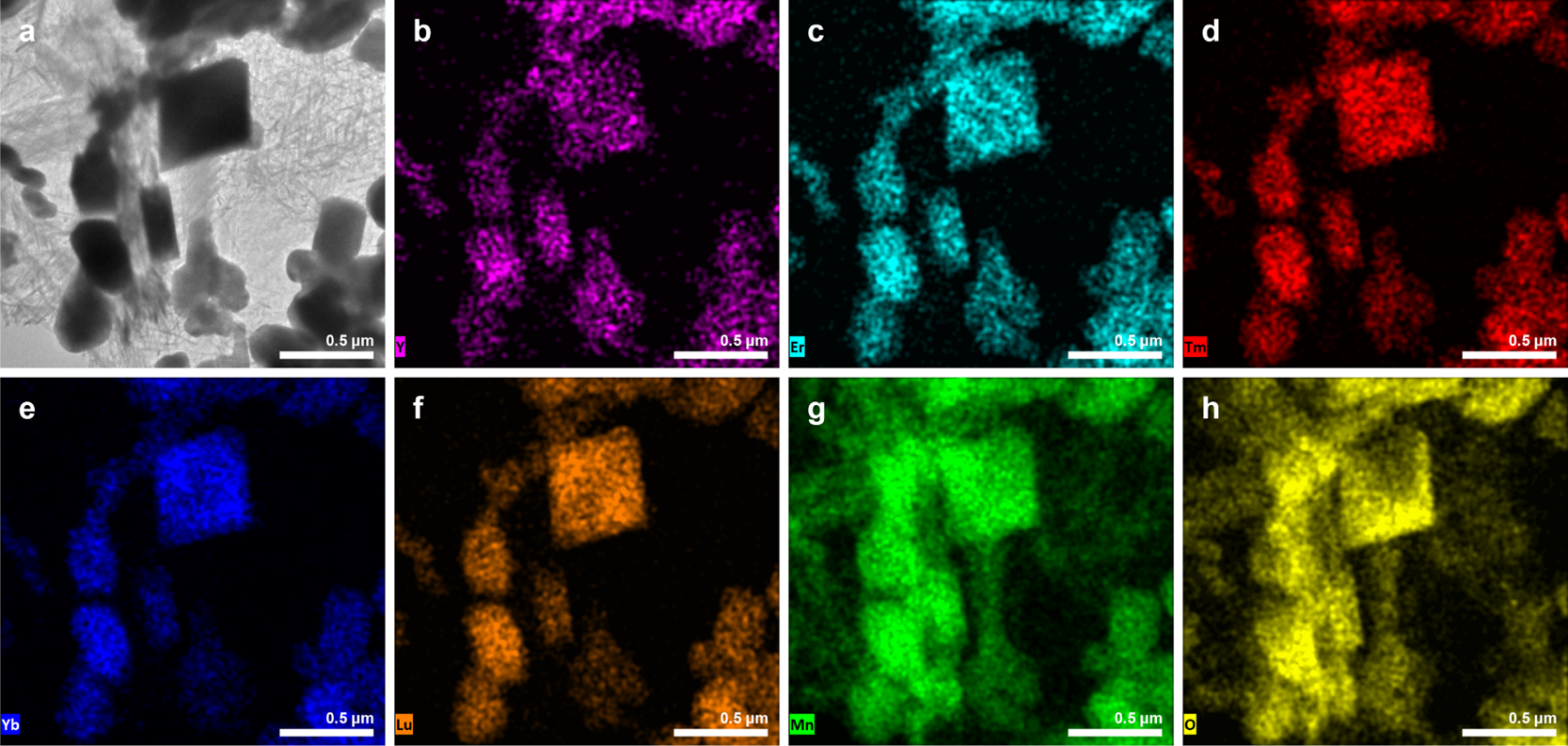
STEM image (a) and elemental mapping of the Y_0.4_Er_0.4_Tm_0.4_Yb_0.4_Lu_0.4_Mn_2_O_7_ solid solution demonstrating the distribution of Y (b); Er (c); Tm (d); Yb (e); Lu (f); Mn (g); and O (h).

The STEM image of the pyrochlore solid solution and EDS mapping are given in Figure 9. The data support the results of XRD and reveal that all A-type cations are evenly distributed in the material as there are no visible regions with high concentrations of some rare-earth elements and complete absence of others, which also confirms the formation of the solid solution. Nevertheless, it was recently shown that the synthesis of high-entropy oxides by flux routes may result in different compositions of the final product compared to the ratio of starting materials, which might be due to the different solubilities or differences in reagent particle sizes.^47^ The results of EDX analysis demonstrated that the percentage of Y was lower compared to that of other lanthanides, while the content of the rest of the A-site cations was comparable. The small amount of needle-like particles, which were also seen in the SEM image (inset of Figure 8), was confirmed to be a manganese oxide since only Mn and O were detected in this area.

Finally, analysis of the magnetic susceptibility data lead to a μ_eff_ of 5.85 μ_B_, which is slightly smaller than the average of the five metals (8.32 μ_eff_) (Figures S28–S29). Summarizing the obtained results, it can be concluded that rare-earth manganese pyrochlores and their solid solutions can be successfully synthesized by the presented molten salt method at ambient pressure in an air atmosphere. Further investigation should be directed toward *in situ* analysis in order to determine the mechanism of the formation of the described R_2_Mn_2_O_7_.

## 4 Conclusions

A low-temperature, simple, time- and cost-effective approach for the preparation of metastable R_2_Mn_2_O_7_ pyrochlores by the molten salt method was demonstrated. The suggested synthetic route requires neither high pressure and high temperature nor oxygen flow, which are traditionally used for the preparation of this class of materials. A series of pyrochlores (R = Y, Ho–Lu) was successfully synthesized at 700 °C at ambient pressure in an air atmosphere, which allows for transfer of this synthetic procedure to any laboratory. All synthesized pyrochlores demonstrated ferromagnetic behavior at low temperature, and the magnetic properties were in good agreement with those of high-pressure synthesized analogues. Moreover, we showed the phase-selective synthesis of o-YMnO_3_, h-YMnO_3_, and Y_2_Mn_2_O_7_ by a simple variation of annealing temperature and precursors-to-chlorides ratio. Finally, the versatility of the method was demonstrated by the preparation of a mixed-rare earth Y_0.4_Er_0.4_Tm_0.4_Yb_0.4_Lu_0.4_Mn_2_O_7_ solid solution.

## Data Availability

Additional data can be found in the Supporting Information file.

## Supporting Information Available

XRD patterns of the synthesis products for other precursors and Ln = Dy; Rietveld refinement data for Ln_2_Mn_2_O_7_ for Ln = Y, Ho–Lu; additional XPS spectra; magnetic data including *M*(*H*), ZFC/FCW, and Curie–Weiss plots for each pyrochlore; Rietveld refinement for the high-entropy pyrochlore; comparison of the PXRD for all the pyrochlores; and ZFC/FCW and Curie–Weiss plots for the high-entropy pyrochlore (PDF)

## References

[ref1] Xu L.; Meng J.; Liu Q.; Meng J.; Liu X.; Zhang H. Strategy for achieving multiferroic E-type magnetic order in orthorhombic manganites RMnO3 (R = La–Lu). Phys. Chem. Chem. Phys. 2020, 22, 4905–4915. 10.1039/c9cp06275k. 32073064

[ref2] Li M.; Tan H.; Duan W. Hexagonal rare-earth manganites and ferrites: a review of improper ferroelectricity, magnetoelectric coupling, and unusual domain walls. Phys. Chem. Chem. Phys. 2020, 22, 14415–14432. 10.1039/d0cp02195d. 32584340

[ref3] Goto T.; Kimura T.; Lawes G.; Ramirez A. P.; Tokura Y. Ferroelectricity and Giant Magnetocapacitance in Perovskite Rare-Earth Manganites. Phys. Rev. Lett. 2004, 92, 257201 10.1103/physrevlett.92.257201. 15245056

[ref4] Cai Y. Q.; Jiao Y. Y.; Cui Q.; Cai J. W.; Li Y.; Wang B. S.; Fernández-Díaz M. T.; McGuire M. A.; Yan J. Q.; Alonso J. A.; Cheng J. G. Giant reversible magnetocaloric effect in the pyrochlore Er_2_Mn_2_O_7_ due to a cooperative two-sublattice ferromagnetic order. Phys. Rev. Mater. 2017, 1, 064408 10.1103/physrevmaterials.1.064408.

[ref5] Cui Y.; Peng H.; Wu S.; Wang R.; Wu T. Complementary Charge Trapping and Ionic Migration in Resistive Switching of Rare-Earth Manganite TbMnO3. ACS Appl. Mater. Interfaces 2013, 5, 1213–1217. 10.1021/am301769f. 23343576

[ref6] Han H.; Song S.; Lee J. H.; Kim K. J.; Kim G.-W.; Park T.; Jang H. M. Switchable Photovoltaic Effects in Hexagonal Manganite Thin Films Having Narrow Band Gaps. Chem. Mater. 2015, 27, 7425–7432. 10.1021/acs.chemmater.5b03408.

[ref7] González-Castaño M.; de Miguel J. N.; Penkova A.; Centeno M. A.; Odriozola J. A.; Arellano-Garcia H. Ni/YMnO_3_ perovskite catalyst for CO_2_ methanation. Appl. Mater. Today 2021, 23, 101055 10.1016/j.apmt.2021.101055.

[ref8] Otomo M.; Hasegawa T.; Asakura Y.; Yin S. Remarkable Effects of Lanthanide Substitution for the Y-Site on the Oxygen Storage/Release Performance of YMnO_3+δ_. ACS Appl. Mater. Interfaces 2021, 13, 31691–31698. 10.1021/acsami.1c06880. 34185497

[ref9] Todd P. K.; McDermott M. J.; Rom C. L.; Corrao A. A.; Denney J. J.; Dwaraknath S. S.; Khalifah P. G.; Persson K. A.; Neilson J. R. Selectivity in Yttrium Manganese Oxide Synthesis via Local Chemical Potentials in Hyperdimensional Phase Space. J. Am. Chem. Soc. 2021, 143, 15185–15194. 10.1021/jacs.1c06229. 34491732

[ref10] Todd P. K.; Smith A. M. M.; Neilson J. R. Yttrium Manganese Oxide Phase Stability and Selectivity Using Lithium Carbonate Assisted Metathesis Reactions. Inorg. Chem. 2019, 58, 15166–15174. 10.1021/acs.inorgchem.9b02075. 31682435

[ref11] Todd P. K.; Wustrow A.; McAuliffe R. D.; McDermott M. J.; Tran G. T.; McBride B. C.; Boeding E. D.; O’Nolan D.; Liu C.-H.; Dwaraknath S. S.; Chapman K. W.; Billinge S. J. L.; Persson K. A.; Huq A.; Veith G. M.; Neilson J. R. Defect-Accommodating Intermediates Yield Selective Low-Temperature Synthesis of YMnO3 Polymorphs. Inorg. Chem. 2020, 59, 13639–13650. 10.1021/acs.inorgchem.0c02023. 32866379

[ref12] Wustrow A.; Huang G.; McDermott M. J.; O’Nolan D.; Liu C.-H.; Tran G. T.; McBride B. C.; Dwaraknath S. S.; Chapman K. W.; Billinge S. J. L.; Persson K. A.; Thornton K.; Neilson J. R. Lowering Ternary Oxide Synthesis Temperatures by Solid-State Cometathesis Reactions. Chem. Mater. 2021, 33, 3692–3701. 10.1021/acs.chemmater.1c00700.

[ref13] Gardner J. S.; Gingras M. J. P.; Greedan J. E. Magnetic pyrochlore oxides. Rev. Mod. Phys. 2010, 82, 53–107. 10.1103/revmodphys.82.53.

[ref14] Reimers J. N.; Greedan J. E.; Kremer R. K.; Gmelin E.; Subramanian M. A. Short-range magnetic ordering in the highly frustrated pyrochlore Y_2_Mn_2_O_7_. Phys. Rev. B: Condens. Matter Mater. Phys. 1991, 43, 3387–3394. 10.1103/physrevb.43.3387. 9997651

[ref15] Karoblis D.; Zarkov A.; Garskaite E.; Mazeika K.; Baltrunas D.; Niaura G.; Beganskiene A.; Kareiva A. Study of gadolinium substitution effects in hexagonal yttrium manganite YMnO_3_. Sci. Rep. 2021, 11, 2875 10.1038/s41598-021-82621-6. 33536490 PMC7859184

[ref16] Marshall K. P.; Eidem S. O.; Småbråten D. R.; Selbach S. M.; Grande T.; Einarsrud M.-A. Hydrothermal synthesis of hexagonal YMnO_3_ and YbMnO_3_ below 250 °C. Dalton Trans. 2021, 50, 9904–9913. 10.1039/d1dt01572a. 34212164

[ref17] Pomjakushina E.; Pomjakushin V.; Rolfs K.; Karpinski J.; Conder K. New Synthesis Route and Magnetic Structure of Tm2Mn2O7 Pyrochlore. Inorg. Chem. 2015, 54, 9092–9097. 10.1021/acs.inorgchem.5b01498. 26332012

[ref18] Todd P. K.; Neilson J. R. Selective Formation of Yttrium Manganese Oxides through Kinetically Competent Assisted Metathesis Reactions. J. Am. Chem. Soc. 2019, 141, 1191–1195. 10.1021/jacs.8b10123. 30624059

[ref19] Gupta S. K.; Mao Y. Recent Developments on Molten Salt Synthesis of Inorganic Nanomaterials: A Review. J. Phys. Chem. C 2021, 125, 6508–6533. 10.1021/acs.jpcc.0c10981.

[ref20] Bain G. A.; Berry J. F. Diamagnetic Corrections and Pascal’s Constants. J. Chem. Educ. 2008, 85, 532 10.1021/ed085p532.

[ref21] Morrison G.; zur Loye H.-C. Simple correction for the sample shape and radial offset effects on SQUID magnetometers: Magnetic measurements on Ln_2_O_3_ (Ln=Gd, Dy, Er) standards. J. Solid State Chem. 2015, 221, 334–337. 10.1016/j.jssc.2014.10.026.

[ref22] Oleaga A.; Salazar A.; Prabhakaran D.; Cheng J. G.; Zhou J. S. Critical behavior of the paramagnetic to antiferromagnetic transition in orthorhombic and hexagonal phases of RMnO_3_ (R=Sm, Tb, Dy, Ho, Er, Tm, Yb, Lu, Y). Phys. Rev. B: Condens. Matter Mater. Phys. 2012, 85, 184425 10.1103/physrevb.85.184425.

[ref23] Chen M.; Hallstedt B.; Gauckler L. J. Thermodynamic assessment of the Mn–Y–O system. J. Alloys Compd. 2005, 393, 114–121. 10.1016/j.jallcom.2004.09.057.

[ref24] Fujinaka H.; Kinomura N.; Koizumi M.; Miyamoto Y.; Kume S. Syntheses and physical properties of pyrochlore-type A2B2O7 (A=Tl,Y; B=Cr,Mn). Mater. Res. Bull. 1979, 14, 1133–1137. 10.1016/0025-5408(79)90207-1.

[ref25] Subramanian M. A.; Torardi C. C.; Johnson D. C.; Pannetier J.; Sleight A. W. Ferromagnetic R_2_Mn_2_O_7_ pyrochlores (R = Dy, Lu, Y). J. Solid State Chem. 1988, 72, 24–30. 10.1016/0022-4596(88)90004-7.

[ref26] Shimakawa Y.; Kubo Y.; Hamada N.; Jorgensen J. D.; Hu Z.; Short S.; Nohara M.; Takagi H. Crystal structure, magnetic and transport properties, and electronic band structure of A2Mn2O7 pyrochlores (A = Y, In, Lu, and Tl). Phys. Rev. B: Condens. Matter Mater. Phys. 1999, 59, 1249–1254. 10.1103/physrevb.59.1249.

[ref27] Shannon R. D. Revised effective ionic radii and systematic studies of interatomic distances in halides and chalcogenides. Acta Crystallogr., Sect. A: Cryst. Phys., Diffr., Theor. Gen. Crystallogr. 1976, 32, 751–767. 10.1107/s0567739476001551.

[ref28] Fateley W. G. Infrared and Raman Selection Rules for Molecular and Lattice Vibrations: The Correlation Method; Krieger Publishing Company, 1972.

[ref29] Brown S.; Gupta H. C.; Alonso J. A.; Martinez-Lope M. J. Vibrational spectra and force field calculation of A2Mn2O7 (A = Y, Dy, Er, Yb) pyrochlores. J. Raman Spectrosc. 2003, 34, 240–243. 10.1002/jrs.982.

[ref30] Subramanian M. A.; Aravamudan G.; Subba Rao G. V. Oxide pyrochlores—A review. Prog. Solid State Chem. 1983, 15, 55–143. 10.1016/0079-6786(83)90001-8.

[ref31] Brown S.; Gupta H. C.; Alonso J. A.; Martínez-Lope M. J. Lattice dynamical study of optical modes in Tl_2_Mn_2_O_7_ and In_2_Mn_2_O_7_ pyrochlores. Phys. Rev. B: Condens. Matter Mater. Phys. 2004, 69, 054434 10.1103/physrevb.69.054434.

[ref32] Ubaldini A.; Carnasciali M. M. Raman characterisation of powder of cubic RE2O3 (RE=Nd, Gd, Dy, Tm, and Lu), Sc2O3 and Y2O3. J. Alloys Compd. 2008, 454, 374–378. 10.1016/j.jallcom.2006.12.067.

[ref33] Post J. E.; McKeown D. A.; Heaney P. J. Raman spectroscopy study of manganese oxides: Tunnel structures. Am. Mineral. 2020, 105, 1175–1190. 10.2138/am-2020-7390.

[ref34] Bugaris D. E.; zur Loye H.-C. Materials Discovery by Flux Crystal Growth: Quaternary and Higher Order Oxides. Angew. Chem., Int. Ed. 2012, 51, 3780–3811. 10.1002/anie.201102676. 22287424

[ref35] Banerjee D.; Nesbitt H. W. XPS study of reductive dissolution of birnessite by oxalate: rates and mechanistic aspects of dissolution and redox processes. Geochim. Cosmochim. Acta 1999, 63, 3025–3038. 10.1016/s0016-7037(99)00230-6.

[ref36] Biesinger M. C.; Payne B. P.; Grosvenor A. P.; Lau L. W. M.; Gerson A. R.; Smart R. S. C. Resolving surface chemical states in XPS analysis of first row transition metals, oxides and hydroxides: Cr, Mn, Fe, Co and Ni. Appl. Surf. Sci. 2011, 257, 2717–2730. 10.1016/j.apsusc.2010.10.051.

[ref37] Galakhov V. R.; Demeter M.; Bartkowski S.; Neumann M.; Ovechkina N. A.; Kurmaev E. Z.; Lobachevskaya N. I.; Mukovskii Y. M.; Mitchell J.; Ederer D. L. Mn 3s exchange splitting in mixed-valence manganites. Phys. Rev. B: Condens. Matter Mater. Phys. 2002, 65, 113102 10.1103/physrevb.65.113102.

[ref38] Ilton E. S.; Post J. E.; Heaney P. J.; Ling F. T.; Kerisit S. N. XPS determination of Mn oxidation states in Mn (hydr)oxides. Appl. Surf. Sci. 2016, 366, 475–485. 10.1016/j.apsusc.2015.12.159.

[ref39] Cui Q.; Wang N. N.; Su N.; Cai Y. Q.; Wang B. S.; Shinmei T.; Irifune T.; Alonso J. A.; Cheng J. G. Large reversible magnetocaloric effect in the ferromagnetic pyrochlores R_2_Mn_2_O_7_ (R = Dy, Ho, Yb). J. Magn. Magn. Mater. 2019, 490, 165494 10.1016/j.jmmm.2019.165494.

[ref40] Greedan J. E.; Raju N. P.; Maignan A.; Simon C.; Pedersen J. S.; Niraimathi A. M.; Gmelin E.; Subramanian M. A. Frustrated pyrochlore oxides, Y_2_Mn_2_O_7_, Ho_2_Mn_2_O_7_, and Yb_2_Mn_2_O_7_: Bulk magnetism and magnetic microstructure. Phys. Rev. B: Condens. Matter Mater. Phys. 1996, 54, 7189 10.1103/PhysRevB.54.7189. 9984341

[ref41] Imamura N.; Karppinen M.; Yamauchi H.; Goodenough J. B. Magnetic properties of R_2_Mn_2_O_7_ pyrochlore rare-earth solid solutions. Phys. Rev. B: Condens. Matter Mater. Phys. 2010, 82, 132407 10.1103/physrevb.82.132407.

[ref42] Subramanian M.; Sleight A. Rare earth pyrochlores. In Handbook on the Physics and Chemistry of Rare Earths; Elsevier, 1993; Vol. 16, pp 225–248.

[ref43] Sarkar A.; Wang Q.; Schiele A.; Chellali M. R.; Bhattacharya S. S.; Wang D.; Brezesinski T.; Hahn H.; Velasco L.; Breitung B. High-Entropy Oxides: Fundamental Aspects and Electrochemical Properties. Adv. Mater. 2019, 31, 1806236 10.1002/adma.201806236. 30838717

[ref44] Pitike K. C.; Macias A.; Eisenbach M.; Bridges C. A.; Cooper V. R. Computationally Accelerated Discovery of High Entropy Pyrochlore Oxides. Chem. Mater. 2022, 34, 1459–1472. 10.1021/acs.chemmater.1c02361.

[ref45] Sarkar A.; Kruk R.; Hahn H. Magnetic properties of high entropy oxides. Dalton Trans. 2021, 50, 1973–1982. 10.1039/d0dt04154h. 33443275

[ref46] Witte R.; Sarkar A.; Kruk R.; Eggert B.; Brand R. A.; Wende H.; Hahn H. High-entropy oxides: An emerging prospect for magnetic rare-earth transition metal perovskites. Phys. Rev. Mater. 2019, 3, 034406 10.1103/physrevmaterials.3.034406.

[ref47] Hines A. T.; Morrison G.; Tisdale H. B.; Smith M. D.; Besmann T. M.; Mofrad A.; Aziziha M.; Booth R. E.; Sun K.; Was G. S.; zur Loye H.-C. Crystallization of A_3_Ln(BO_3_)_2_ (A = Na, K; Ln = Lanthanide) from a Boric Acid Containing Hydroxide Melt: Synthesis and Investigation of Lanthanide Borates as Potential Nuclear Waste Forms. Inorg. Chem. 2022, 61, 11232–11242. 10.1021/acs.inorgchem.2c01301. 35815496

