# Supplementary material for: Low-Temperature Synthesis of Magnetic Pyrochlores (R2Mn2O7, R = Y, Ho–Lu) at Ambient Pressure and Potential for High-Entropy Oxide Synthesis
Source: Inorg Chem. 2023 Jun 26;62(27):10635–44. doi: 10.1021/acs.inorgchem.3c00913 (PMC10336920; doi:10.1021/acs.inorgchem.3c00913)
Supplement: Supplementary file 1 — ic3c00913_si_001.pdf [file ic3c00913_si_001.pdf]

# Supporting information

# Low Temperature Synthesis of Magnetic Pyrochlores ($R_2Mn_2O_7$, R = Y, Ho-Lu) at Ambient Pressure, and Potential for High Entropy Oxide Synthesis

Dovydas Karoblis[1], Orlando C. Stewart, Jr.[2], Priscilla Glaser[2], Salah Eddin El Jamal[2], Agne Kizalaite[1], Tomas Murauskas[1], Aleksej Zarkov[1,*], Aivaras Kareiva[1], Sarah L. Stoll[2,*]

[1]*Institute of Chemistry, Vilnius University, Naugarduko 24, LT-03225 Vilnius, Lithuania*

[2]*Department of Chemistry, Georgetown University, 37th and O Streets NW, Washington, D.C. 20057, United States*

*Authors to whom correspondence should be addressed.

Aleksej Zarkov: e-mail: aleksej.zarkov@chf.vu.lt; Sarah L. Stoll: e-mail: sls55@georgetown.edu

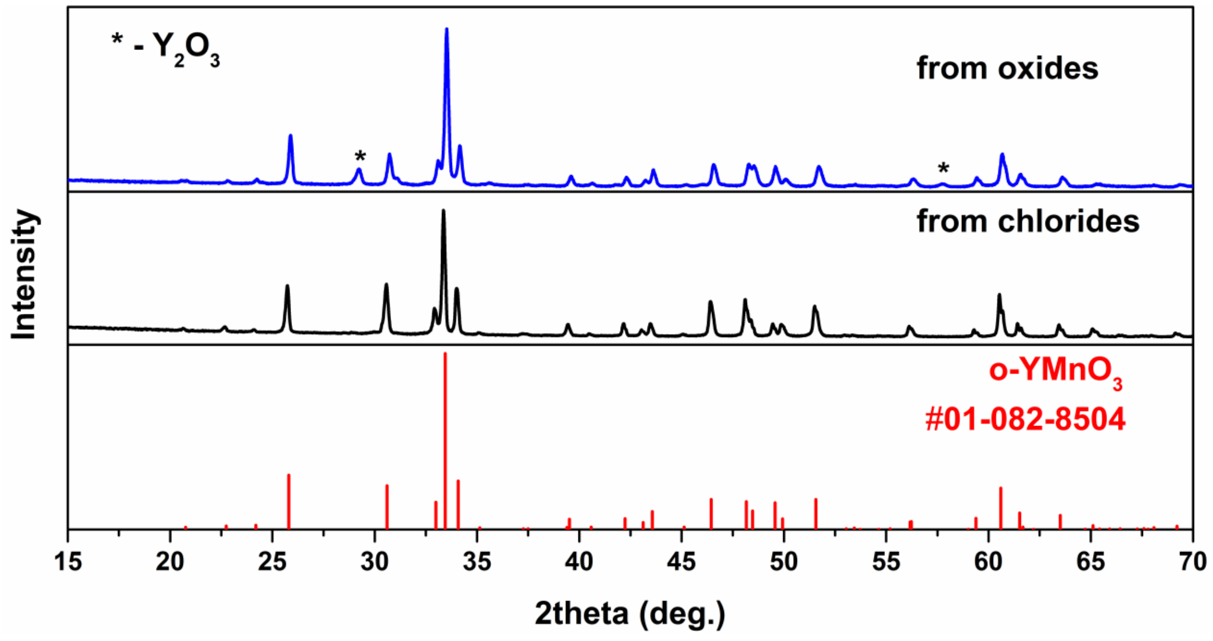


Figure S1. XRD patterns of synthesis products obtained using metal chlorides or metal oxides as starting materials.

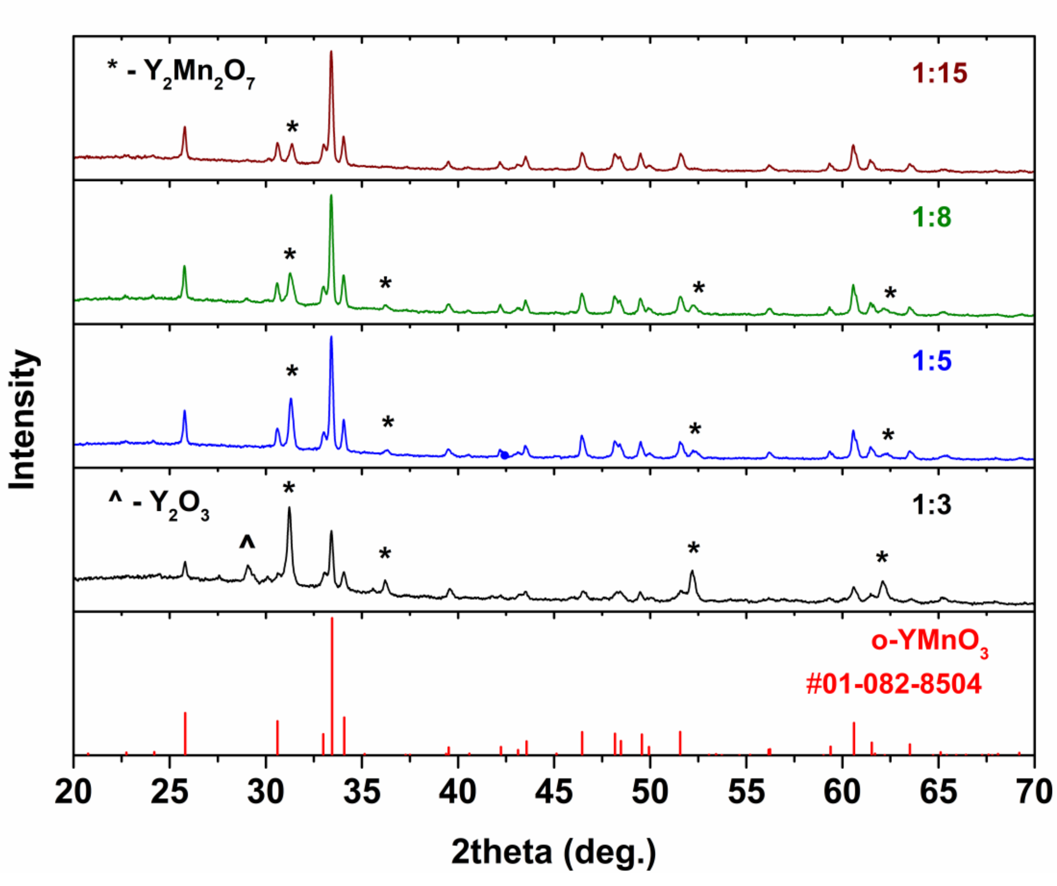


Figure S2. XRD patterns of synthesis products obtained using different ratios of metal nitrates to NaCl and KCl.

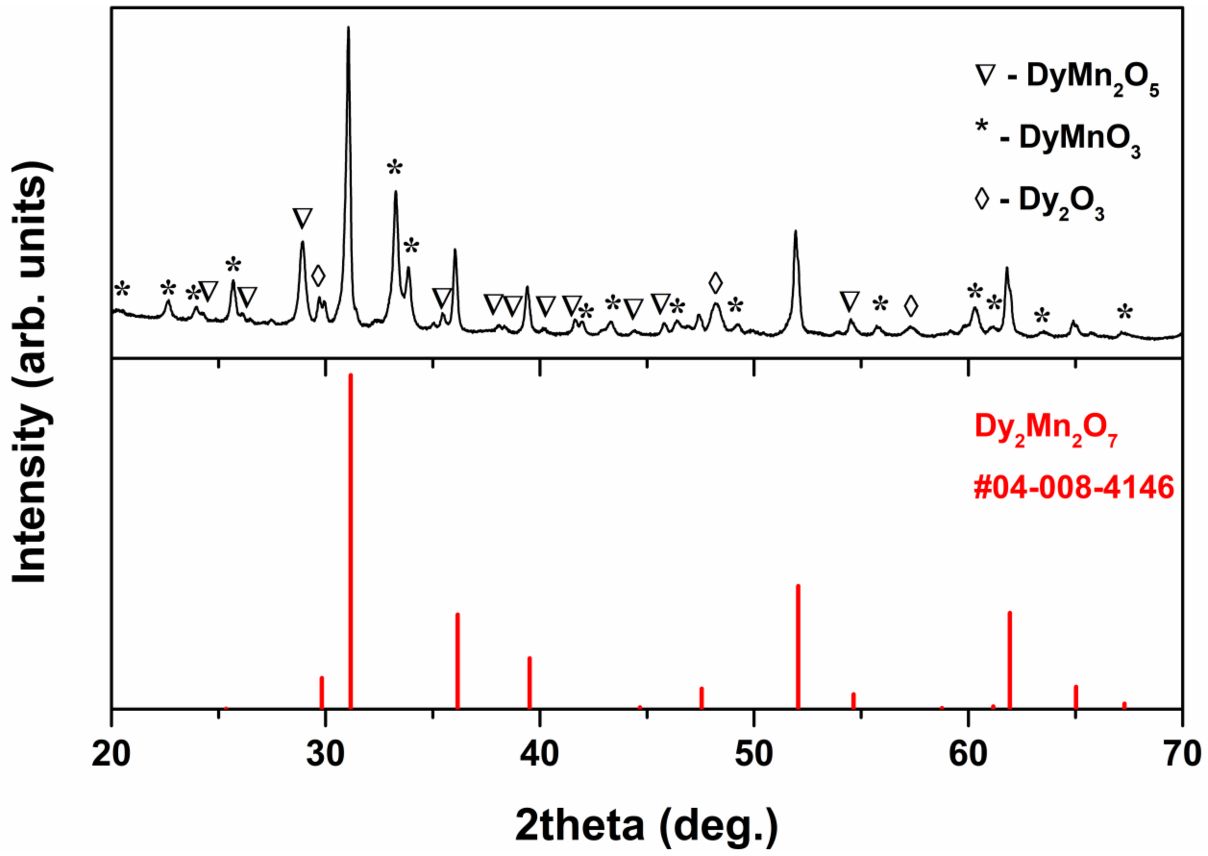


Figure S3. XRD pattern of Dy manganese oxides synthesized under conditions used for the synthesis of other lanthanide pyrochlores.

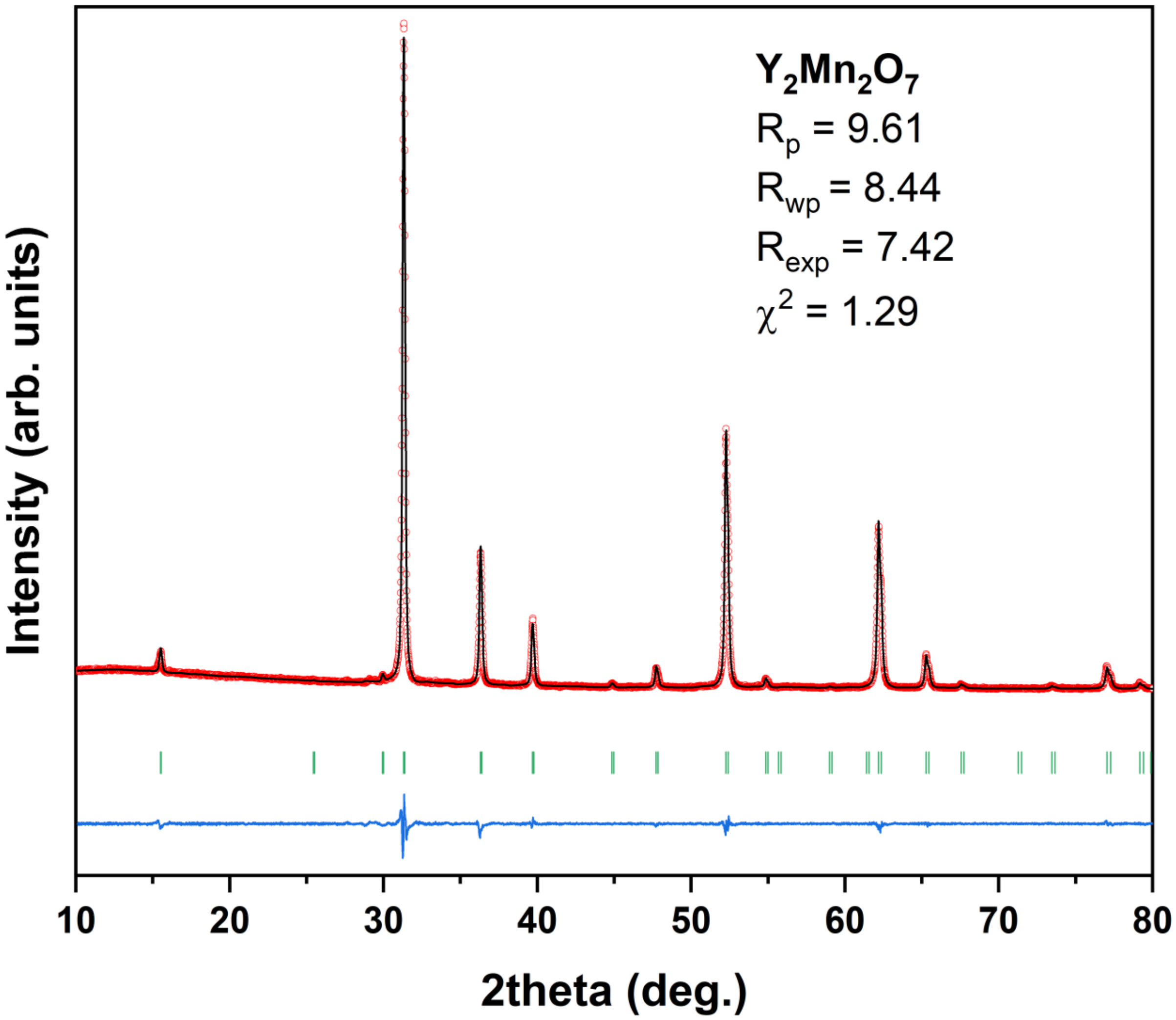


Figure S4. Rietveld refinement data for $Y_2Mn_2O_7$. The red circle symbols and the black solid line represent the experimental and calculated intensities, respectively, and the blue line below is the difference between them. The green tick marks indicate the positions of the Bragg peaks.

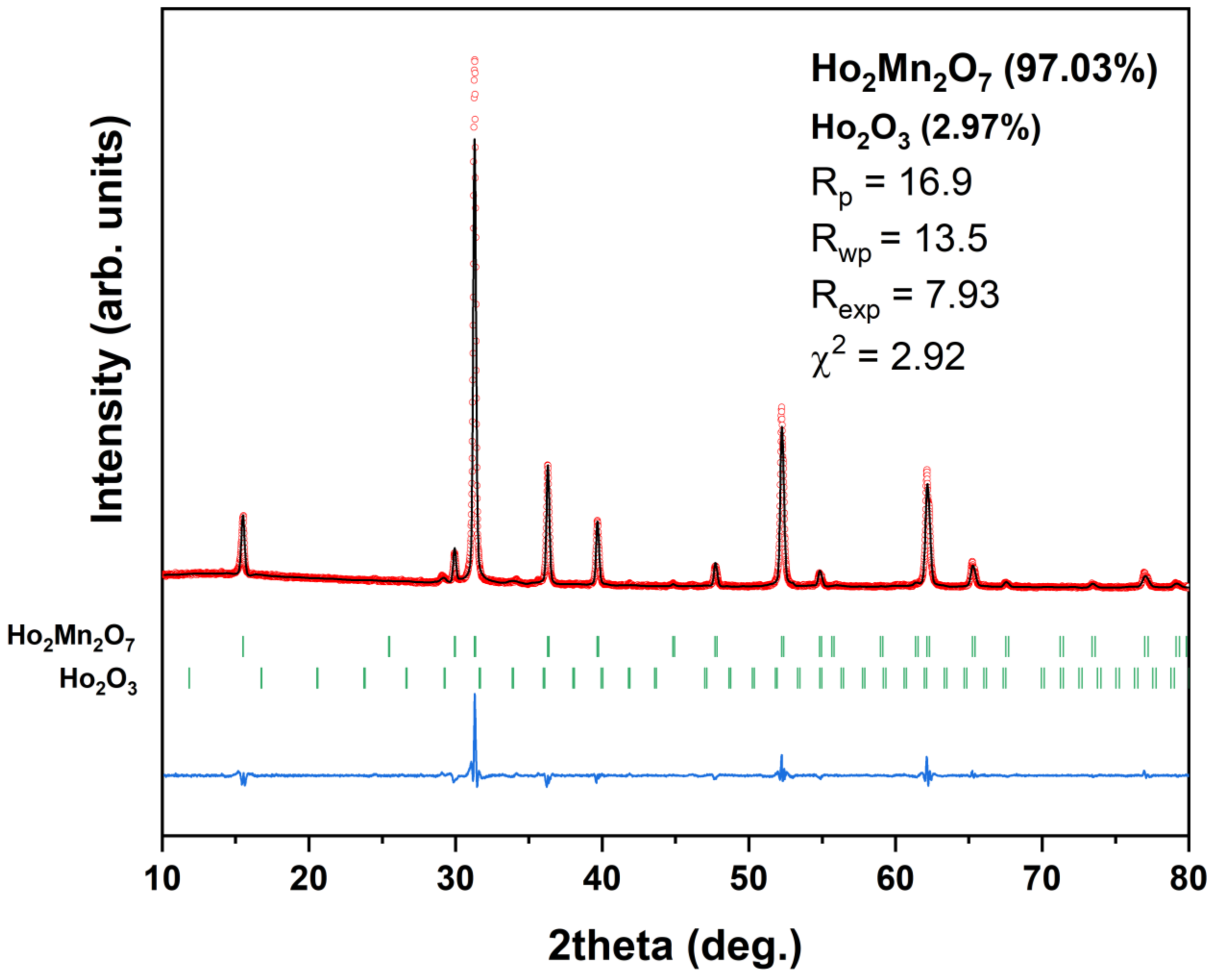


Figure S5. Rietveld refinement data for $Ho_2Mn_2O_7$. The red circle symbols and the black solid line represent the experimental and calculated intensities, respectively, and the blue line below is the difference between them. The green tick marks indicate the positions of the Bragg peaks.

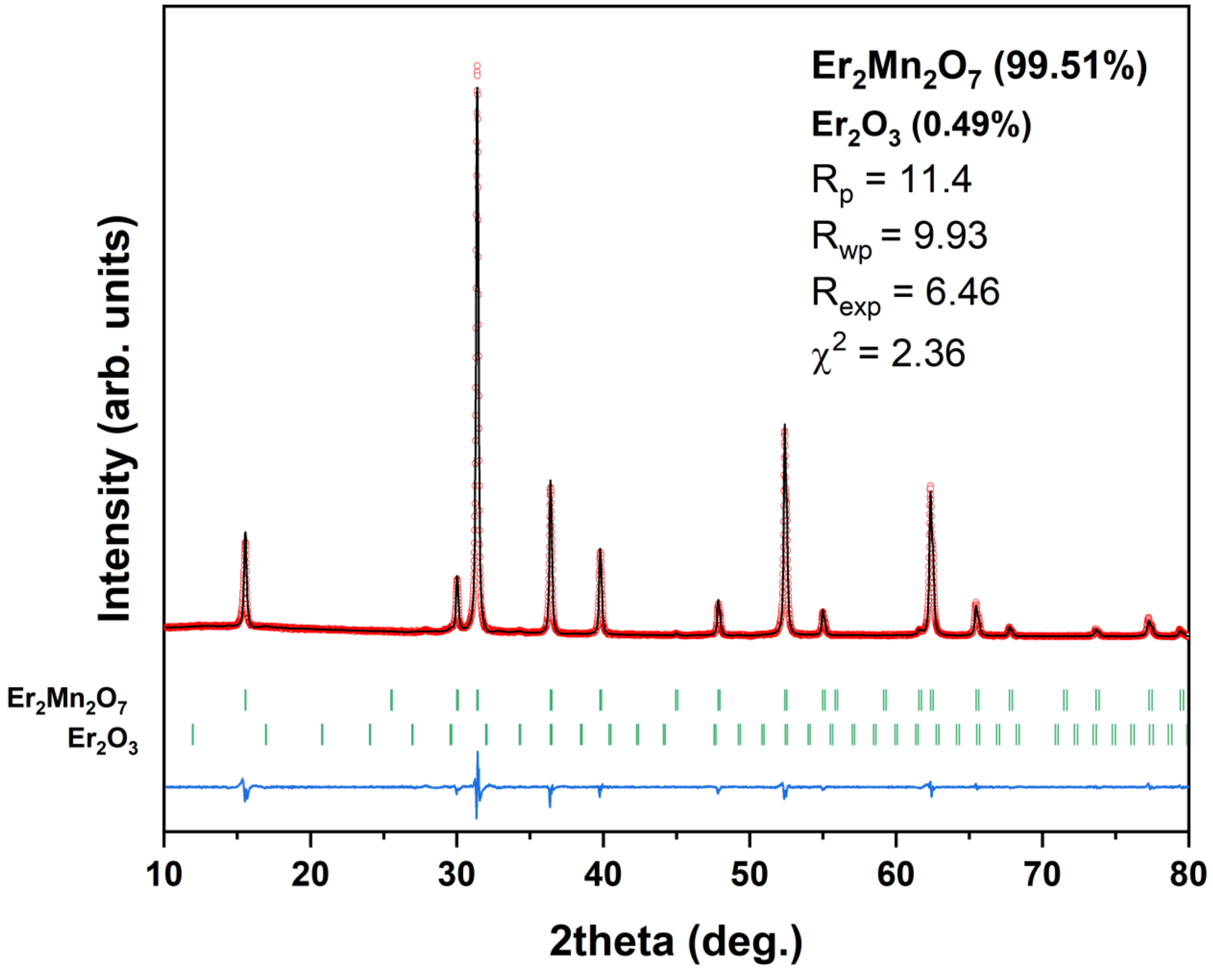


Figure S6. Rietveld refinement data for $Er_2Mn_2O_7$. The red circle symbols and the black solid line represent the experimental and calculated intensities, respectively, and the blue line below is the difference between them. The green tick marks indicate the positions of the Bragg peaks.

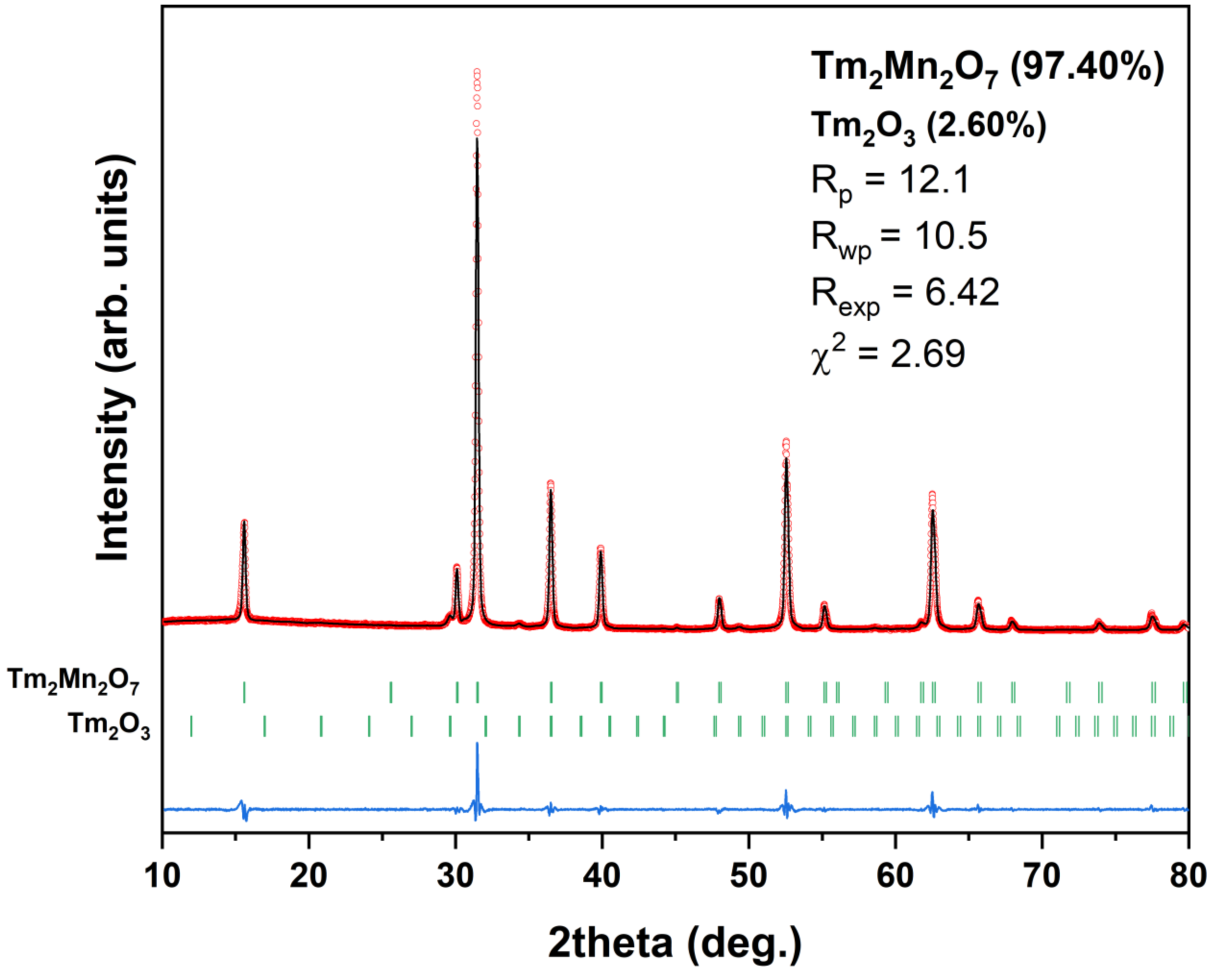


Figure S7. Rietveld refinement data for $Tm_2Mn_2O_7$. The red circle symbols and the black solid line represent the experimental and calculated intensities, respectively, and the blue line below is the difference between them. The green tick marks indicate the positions of the Bragg peaks.

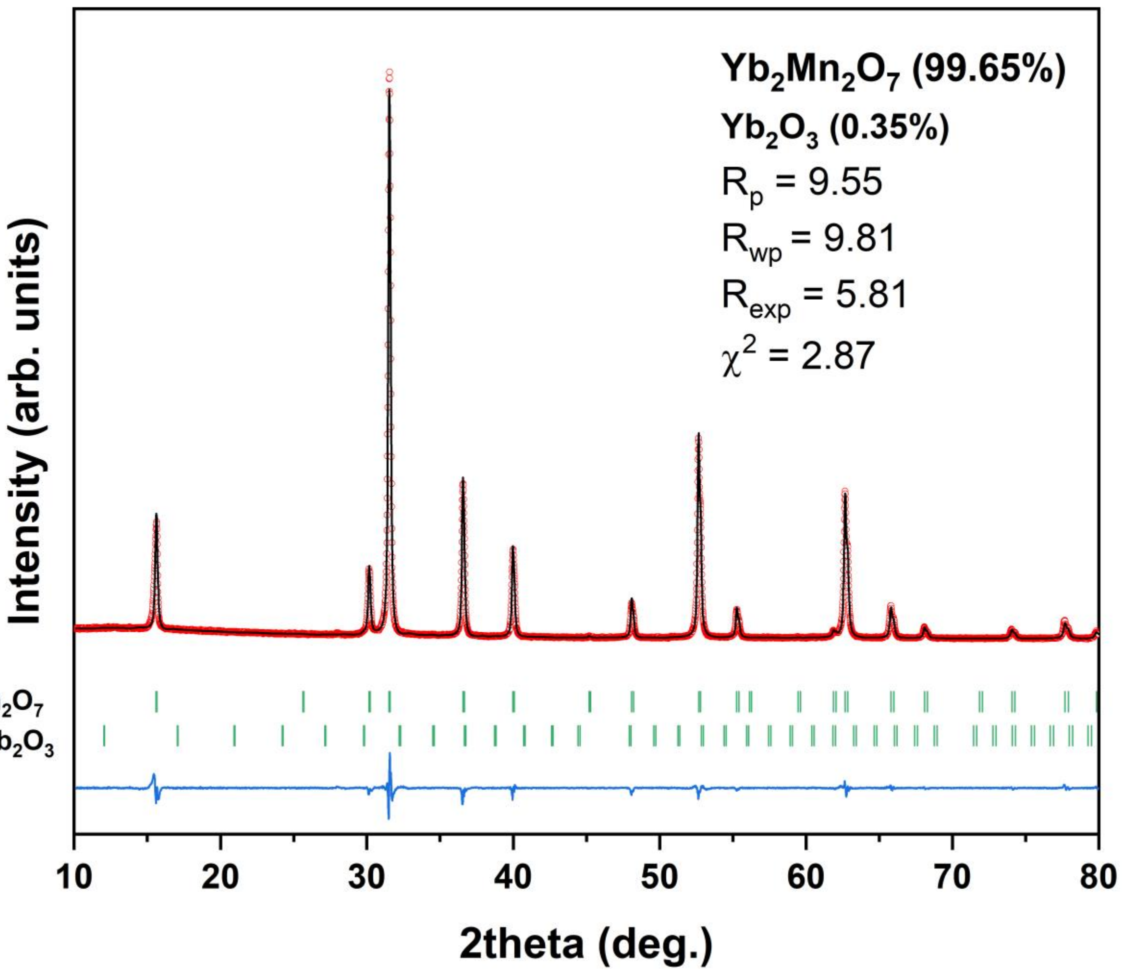


Figure S8. Rietveld refinement data for $Yb_2Mn_2O_7$. The red circle symbols and the black solid line represent the experimental and calculated intensities, respectively, and the blue line below is the difference between them. The green tick marks indicate the positions of the Bragg peaks.

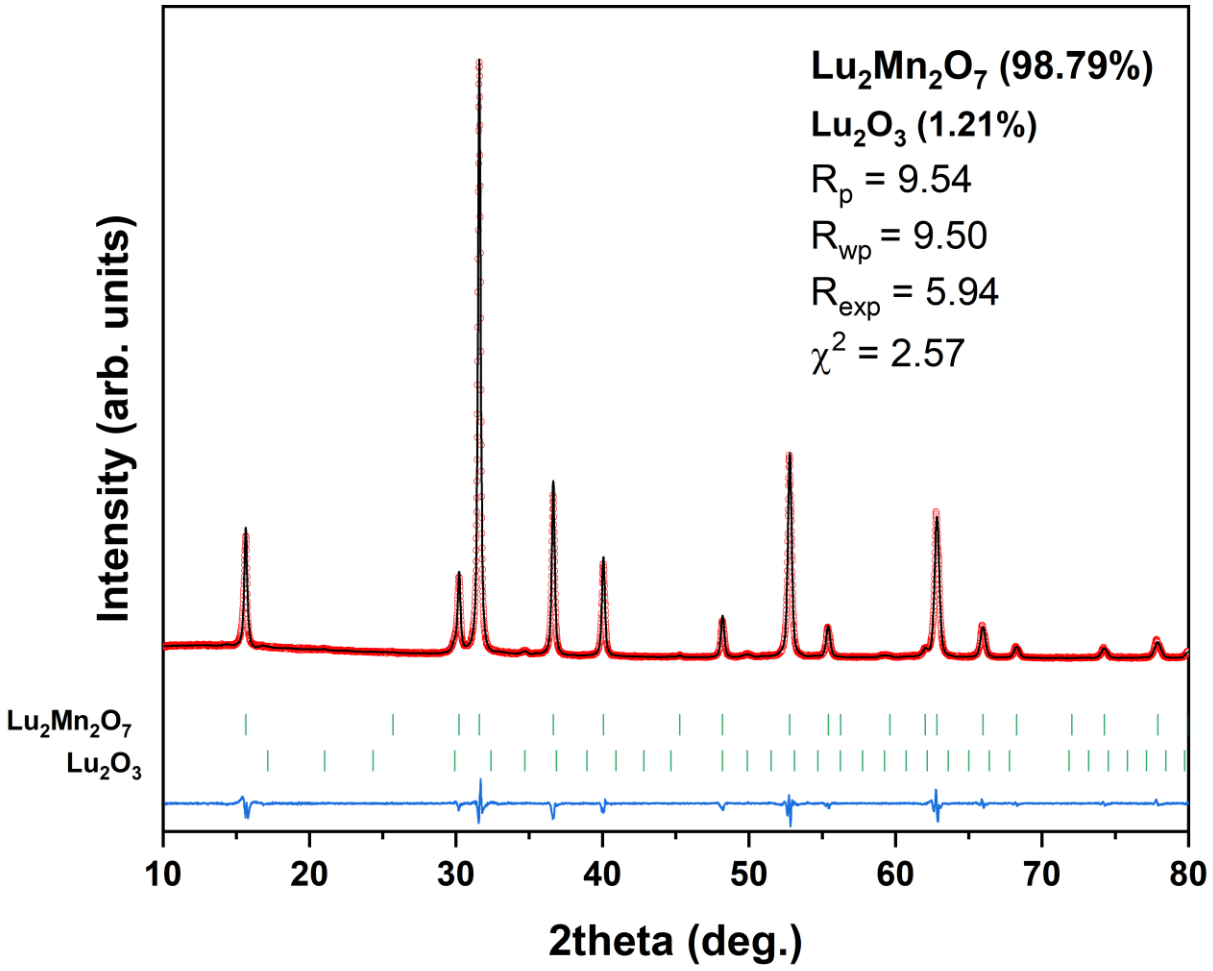


Figure S9. Rietveld refinement data for $Lu_2Mn_2O_7$. The red circle symbols and the black solid line represent the experimental and calculated intensities, respectively, and the blue line below is the difference between them. The green tick marks indicate the positions of the Bragg peaks.

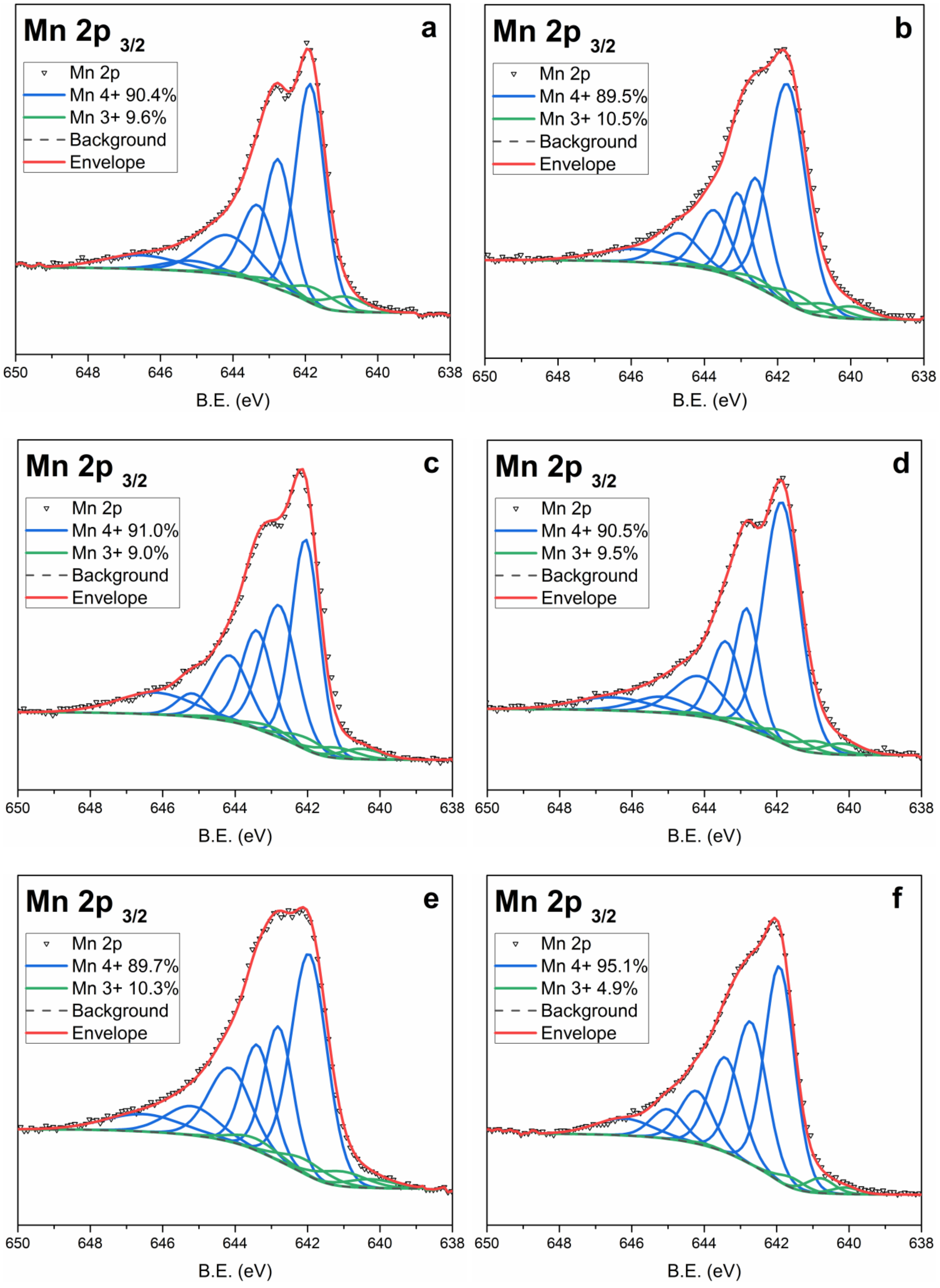


Figure S10. Mn 2p XPS spectra of $Y_2Mn_2O_7$ (a), $Ho_2Mn_2O_7$ (b), $Er_2Mn_2O_7$ (c), $Tm_2Mn_2O_7$ (d), $Yb_2Mn_2O_7$ (e) and $MnO_2$ (f).

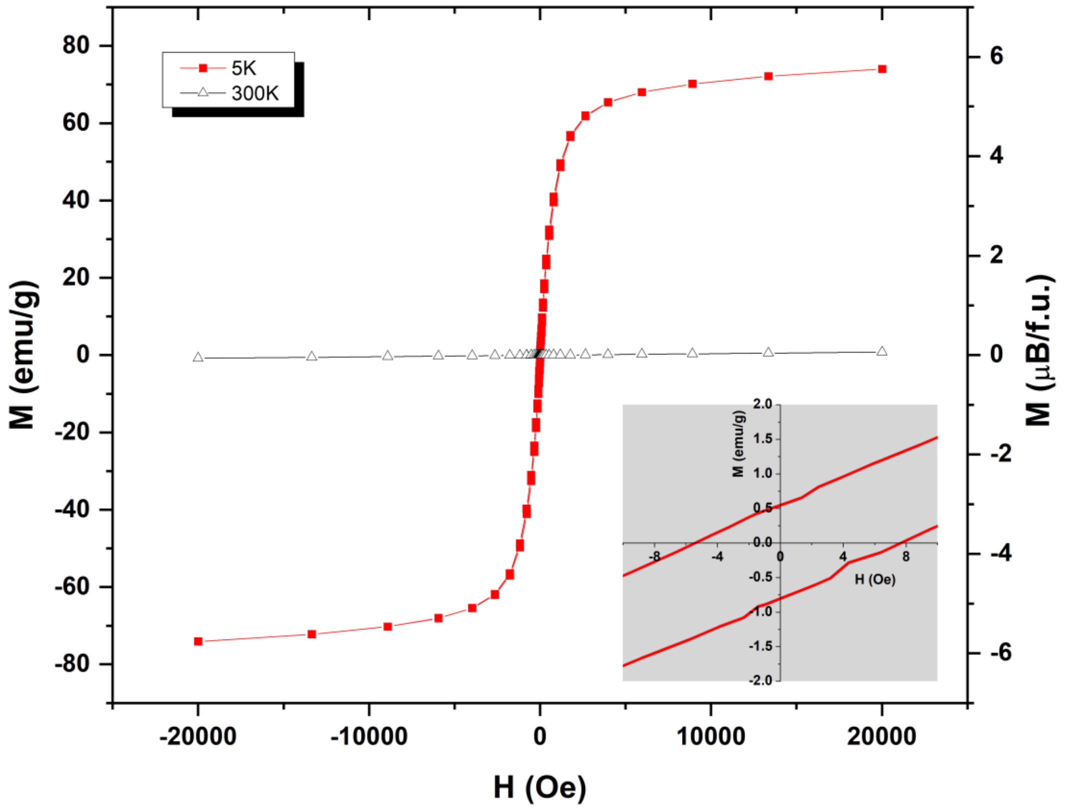


Figure S11. M vs H curves of $Y_2Mn_2O_7$ at 5 K and 300 K. Inset: Zoomed-in curve at 5 K to show coercive field ($H_c$).

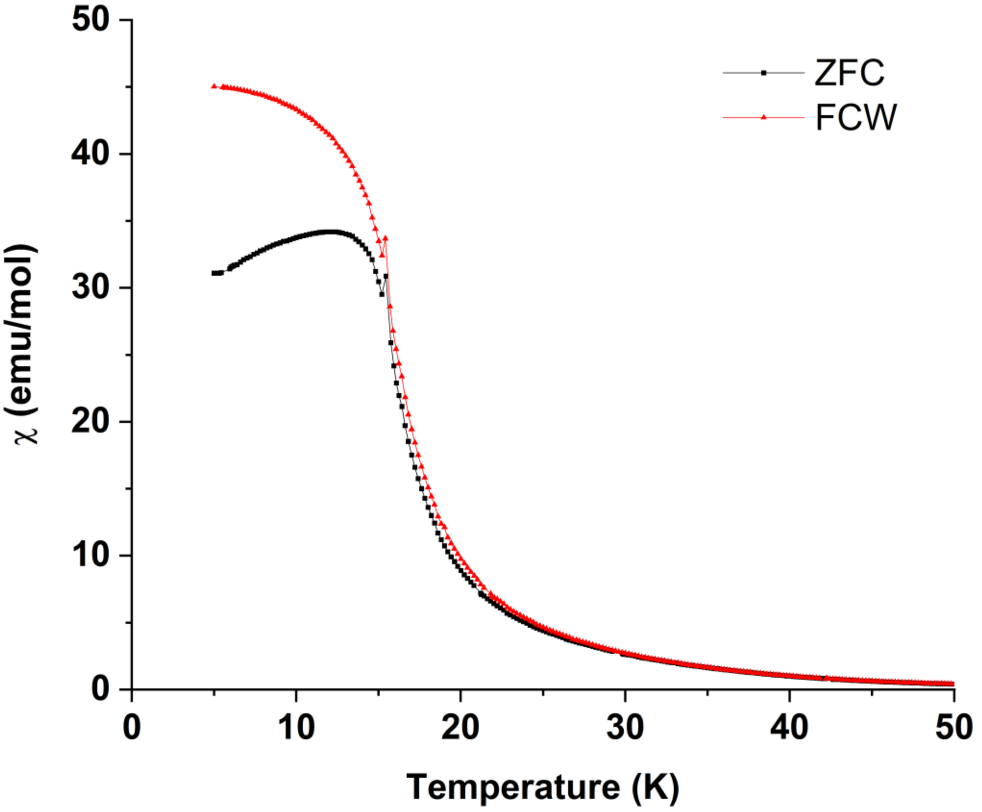


Figure S12. Magnetic susceptibility vs T of $Y_2Mn_2O_7$ at 50 Oe for ZFC and FC data.

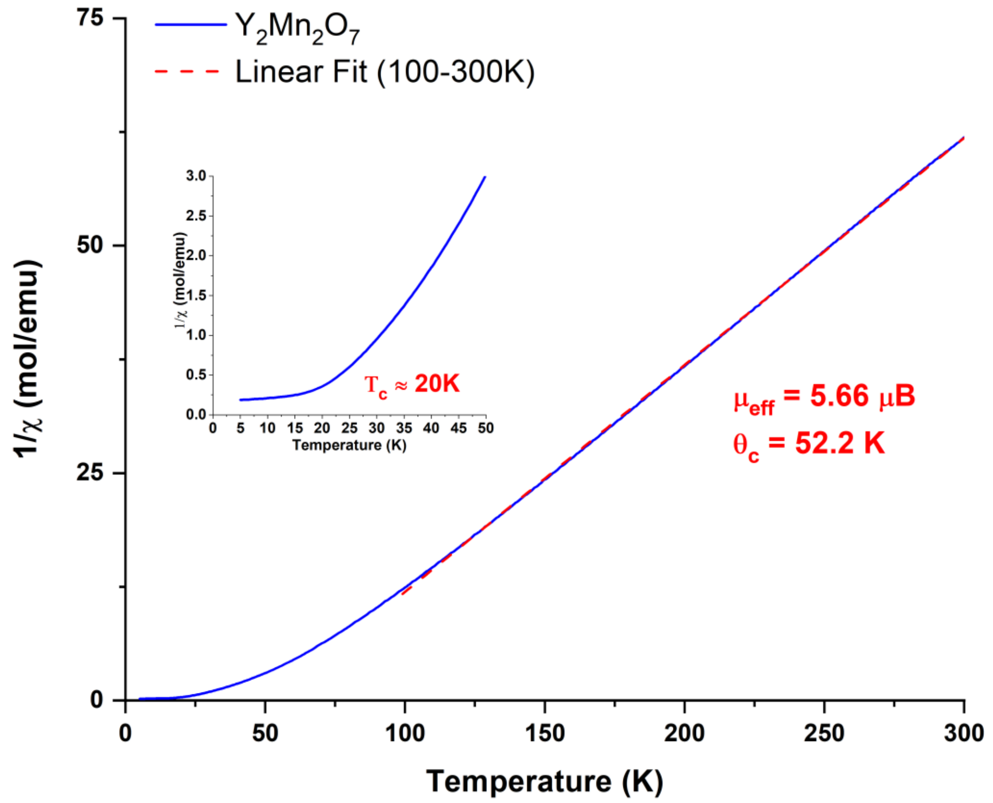


Figure S13. Inverse magnetic susceptibility vs T of $Y_2Mn_2O_7$ at 5000 Oe with Curie-Weiss analysis and linear fit from 100 to 300 K. Inset: Zoomed-in lower T data to show apparent $T_c$.

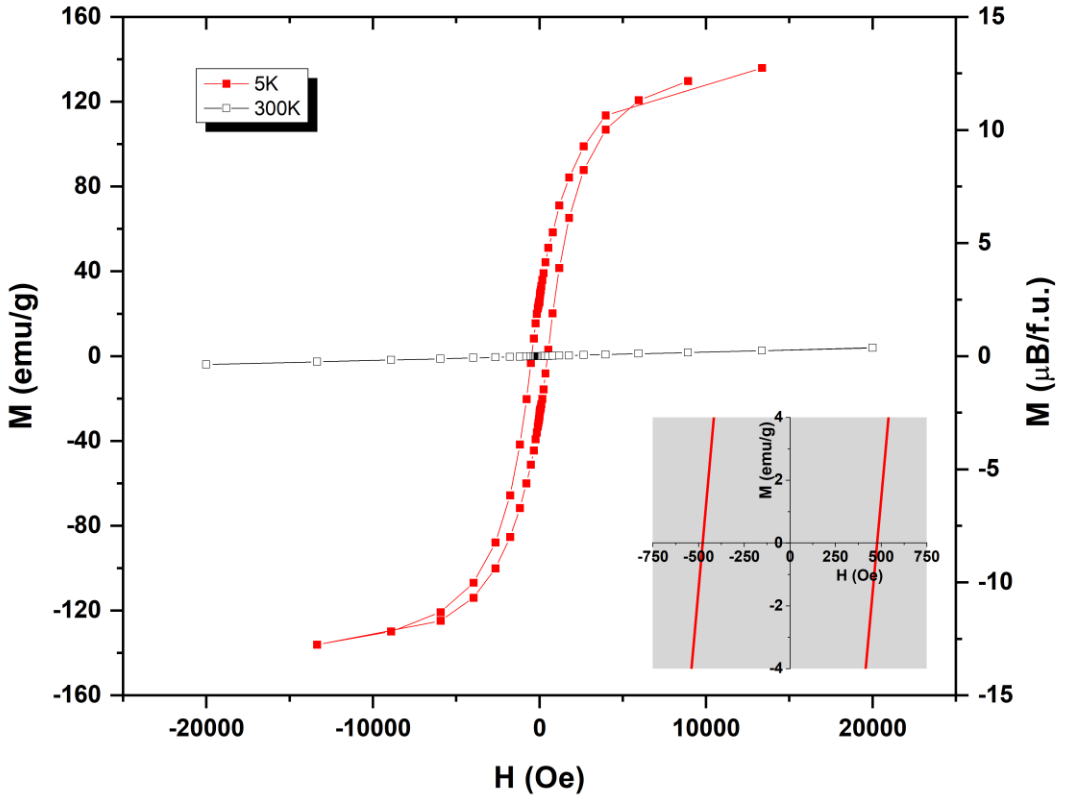


Figure S14. M vs H curves of $Ho_2Mn_2O_7$ at 5 K and 300 K. Inset: Zoomed-in curve at 5 K to show coercive field ($H_c$).

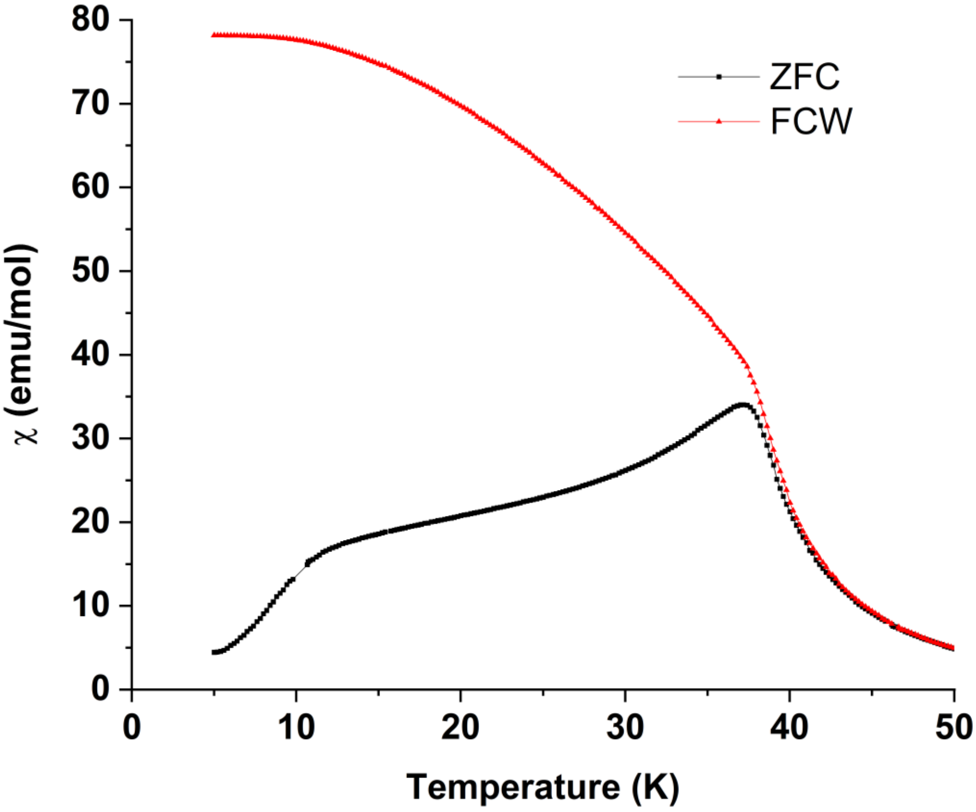


Figure S15. Magnetic susceptibility vs T of $Ho_2Mn_2O_7$ at 50 Oe for ZFC and FC data.

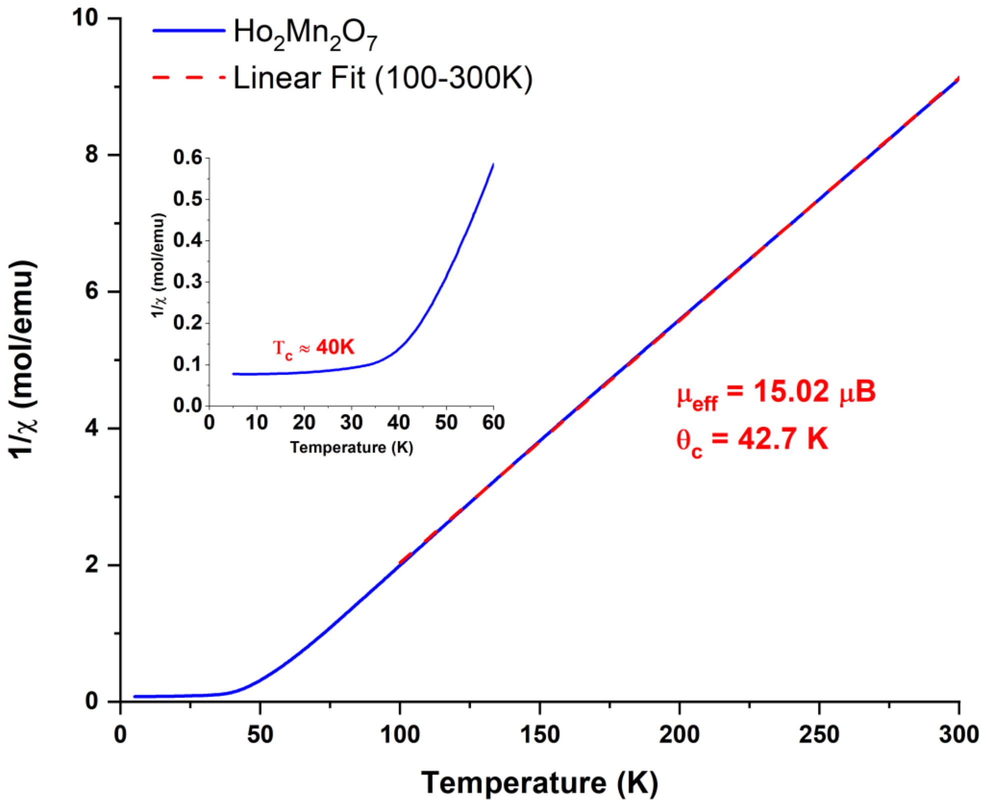


Figure S16. Inverse magnetic susceptibility vs T of $Ho_2Mn_2O_7$ at 5000 Oe with Curie-Weiss analysis and linear fit from 100 to 300 K. Inset: Zoomed-in lower T data to show apparent $T_c$.

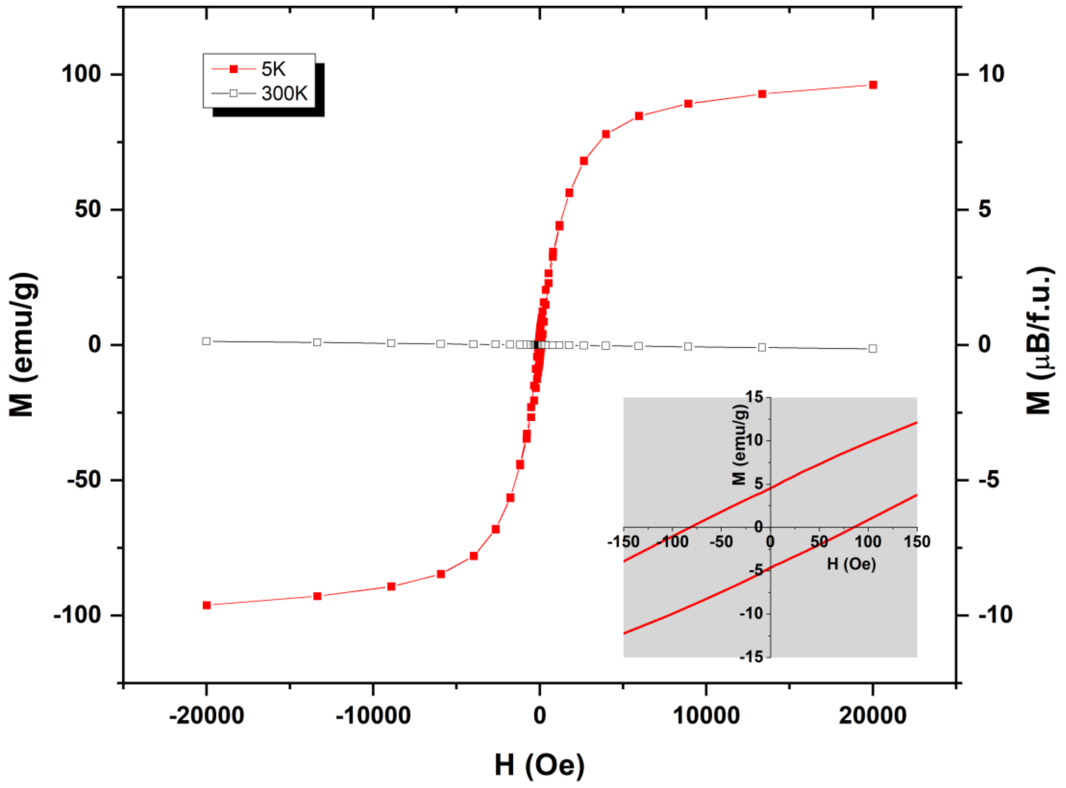


Figure S17. M vs H curves of $Tm_2Mn_2O_7$ at 5 K and 300 K. Inset: Zoomed-in curve at 5 K to show coercive field ($H_c$).

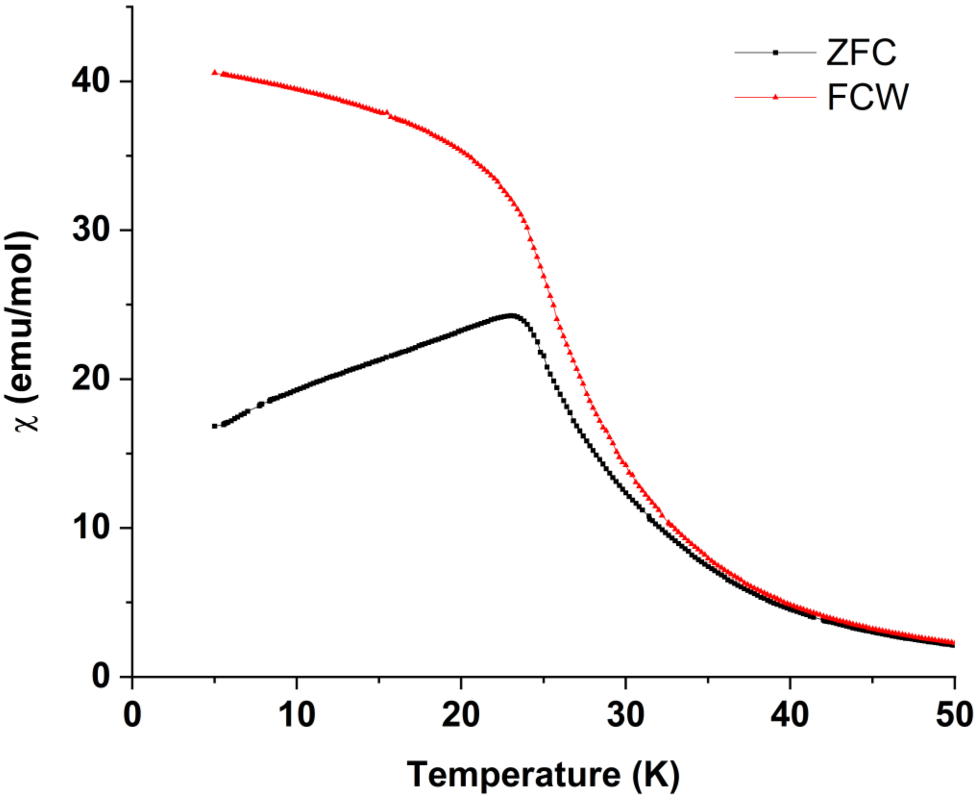


Figure S18. Magnetic susceptibility vs T of $Tm_2Mn_2O_7$ at 50 Oe for ZFC and FC data.

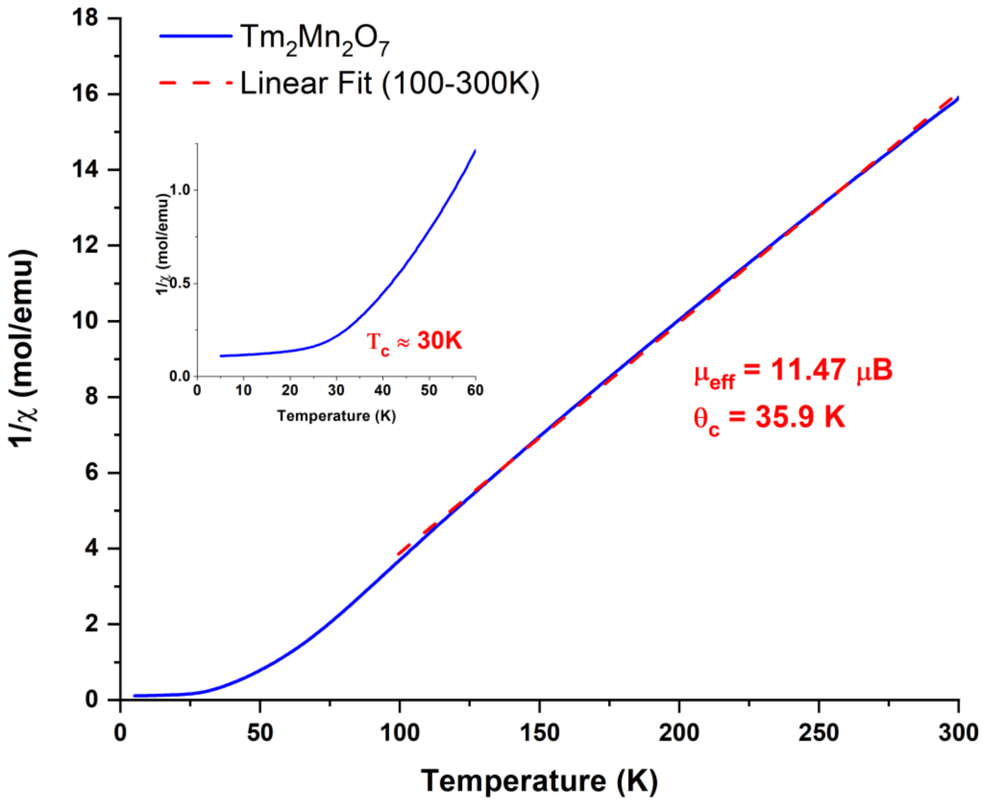


Figure S19. Inverse magnetic susceptibility vs T of $Tm_2Mn_2O_7$ at 5000 Oe with Curie-Weiss analysis and linear fit from 100 to 300 K. Inset: Zoomed-in lower T data to show apparent $T_c$.

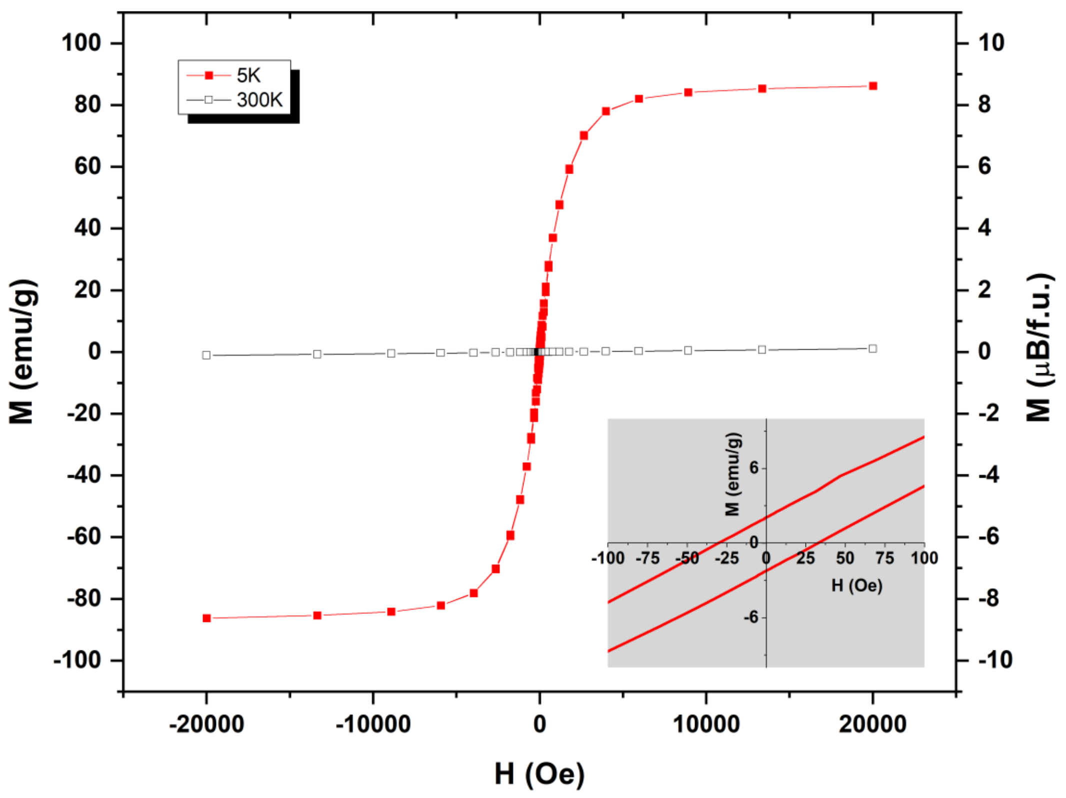


Figure S20. M vs H curves of $Yb_2Mn_2O_7$ at 5 K and 300 K. Inset: Zoomed-in curve at 5 K to show coercive field ($H_c$).

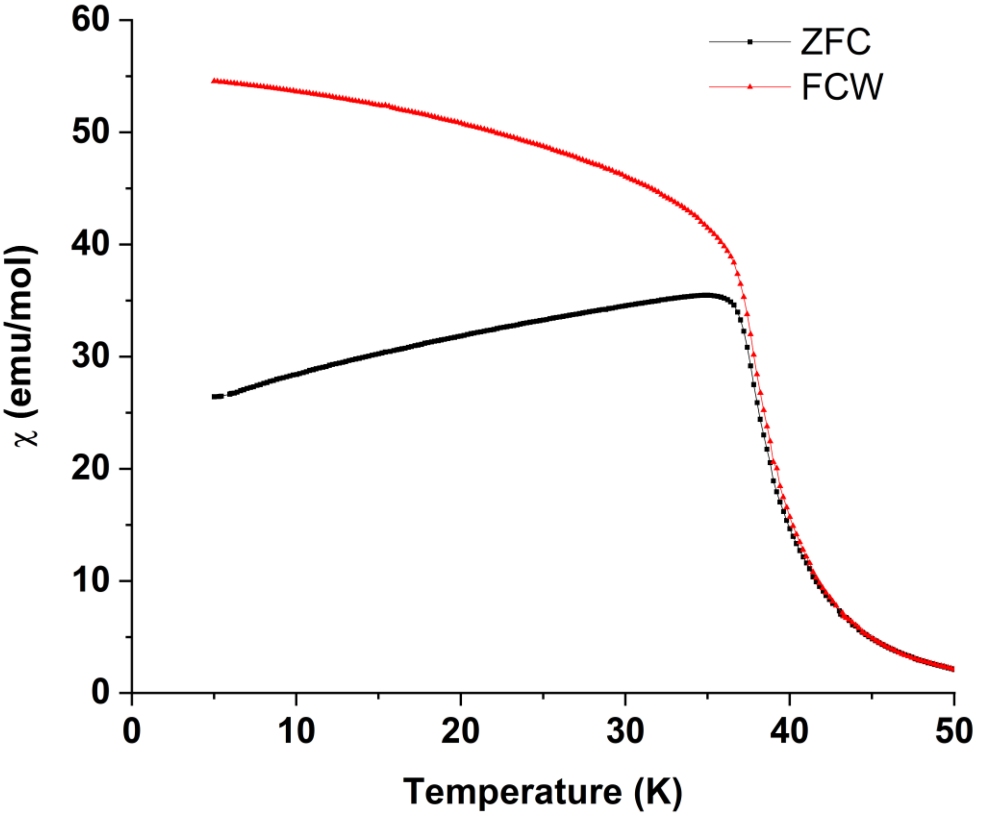


Figure S21. Magnetic susceptibility vs T of $Yb_2Mn_2O_7$ at 50 Oe for ZFC and FC data.

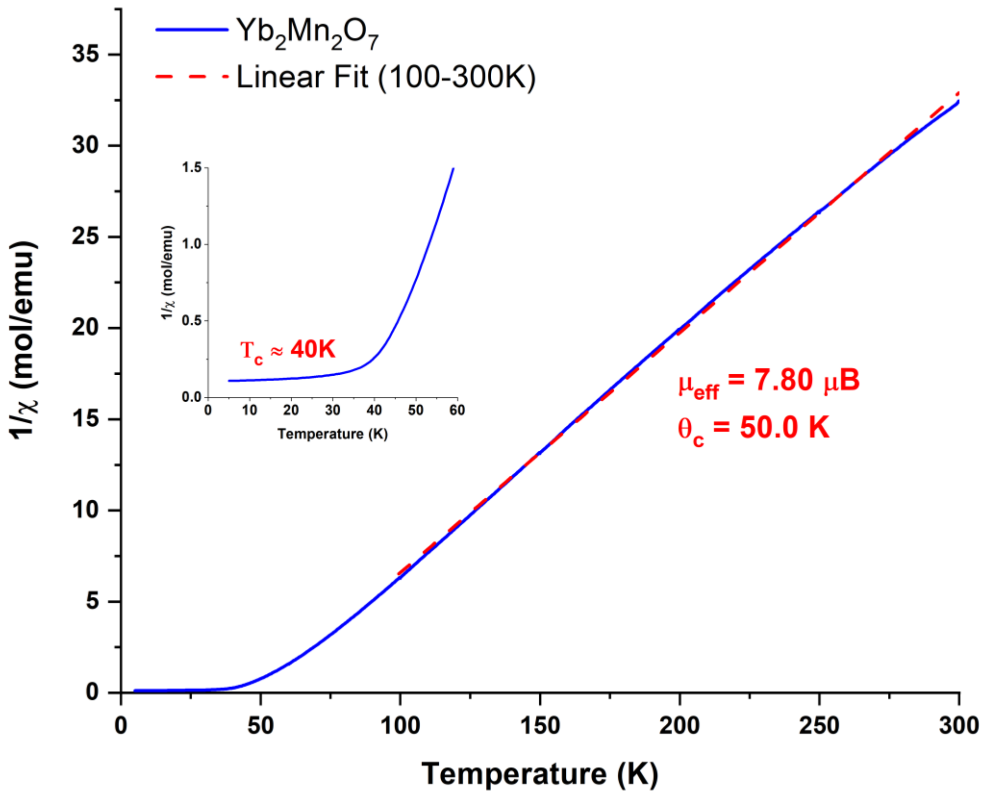


Figure S22. Inverse magnetic susceptibility vs T of $Yb_2Mn_2O_7$ at 5000 Oe with Curie-Weiss analysis and linear fit from 100 to 300 K. Inset: Zoomed-in lower T data to show apparent $T_c$.

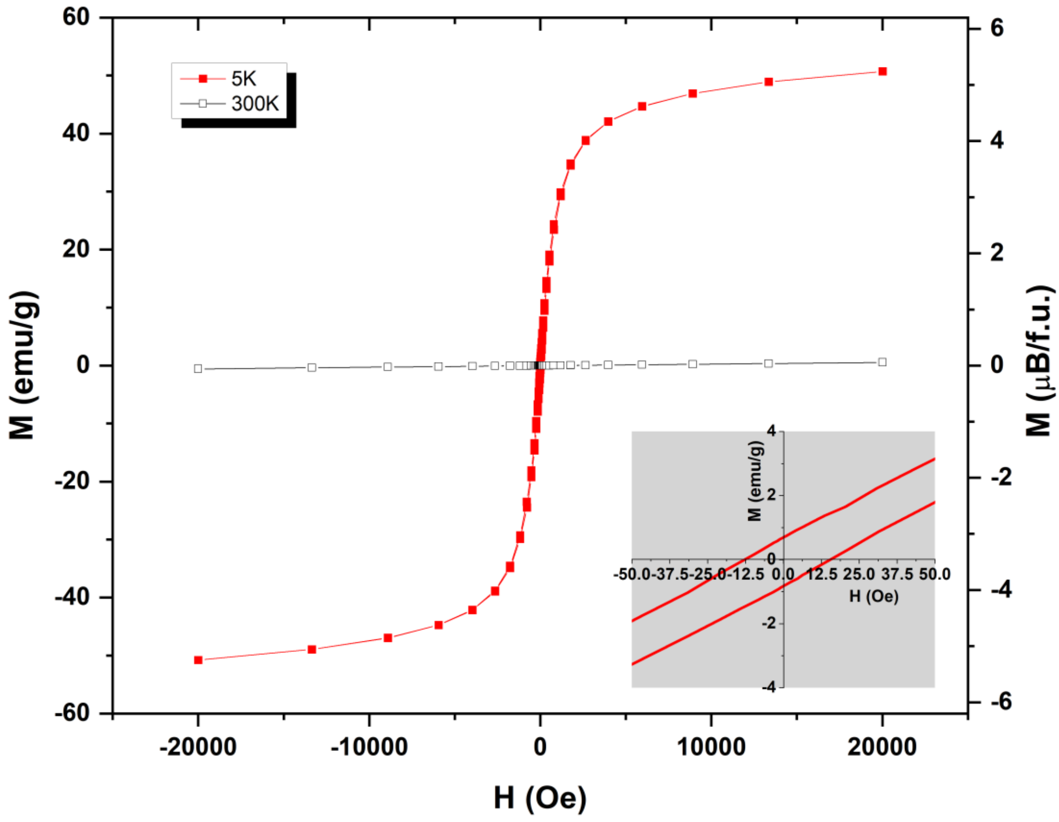


Figure S23. M vs H curves of $Lu_2Mn_2O_7$ at 5 K and 300 K. Inset: Zoomed-in curve at 5 K to show coercive field ($H_c$).

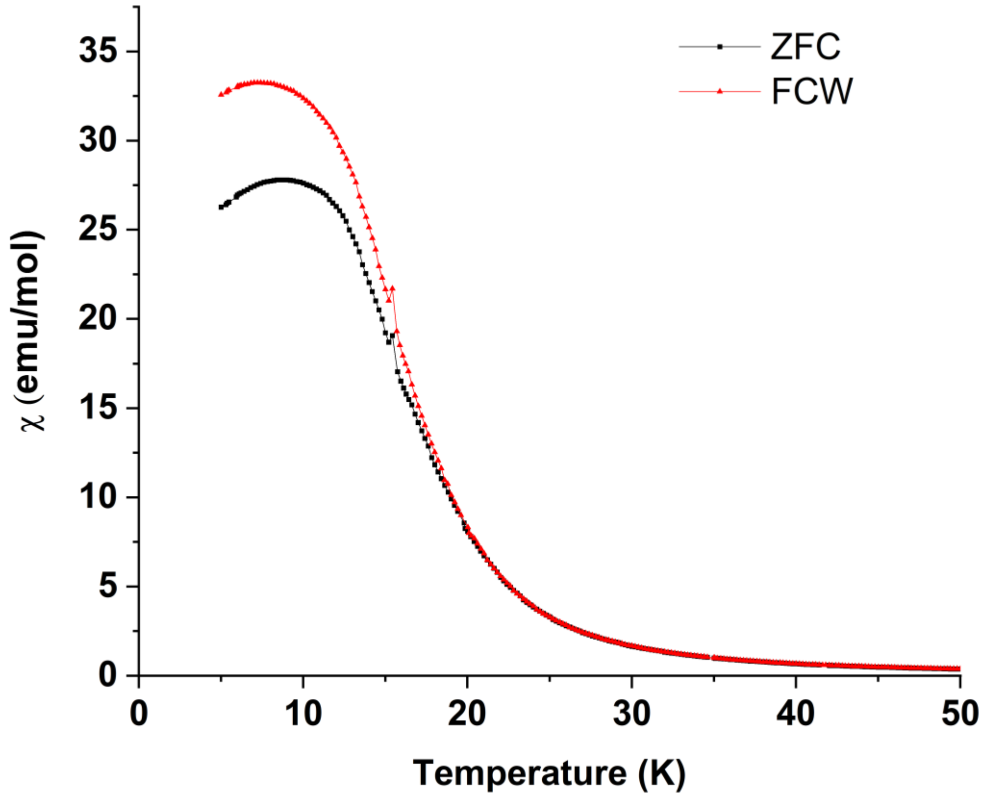


Figure S24. Magnetic susceptibility vs T of $Lu_2Mn_2O_7$ at 50 Oe for ZFC and FC data.

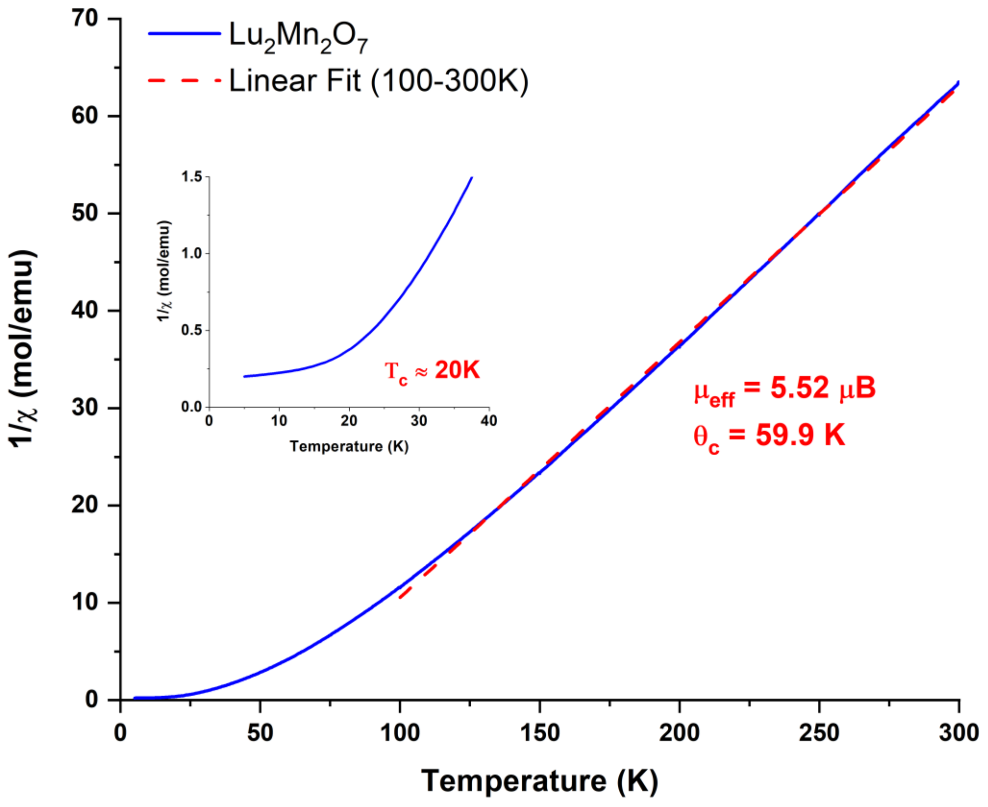


Figure S25. Inverse magnetic susceptibility vs T of $Lu_2Mn_2O_7$ at 5000 Oe with Curie-Weiss analysis and linear fit from 100 to 300 K. Inset: Zoomed-in lower T data to show apparent $T_c$.

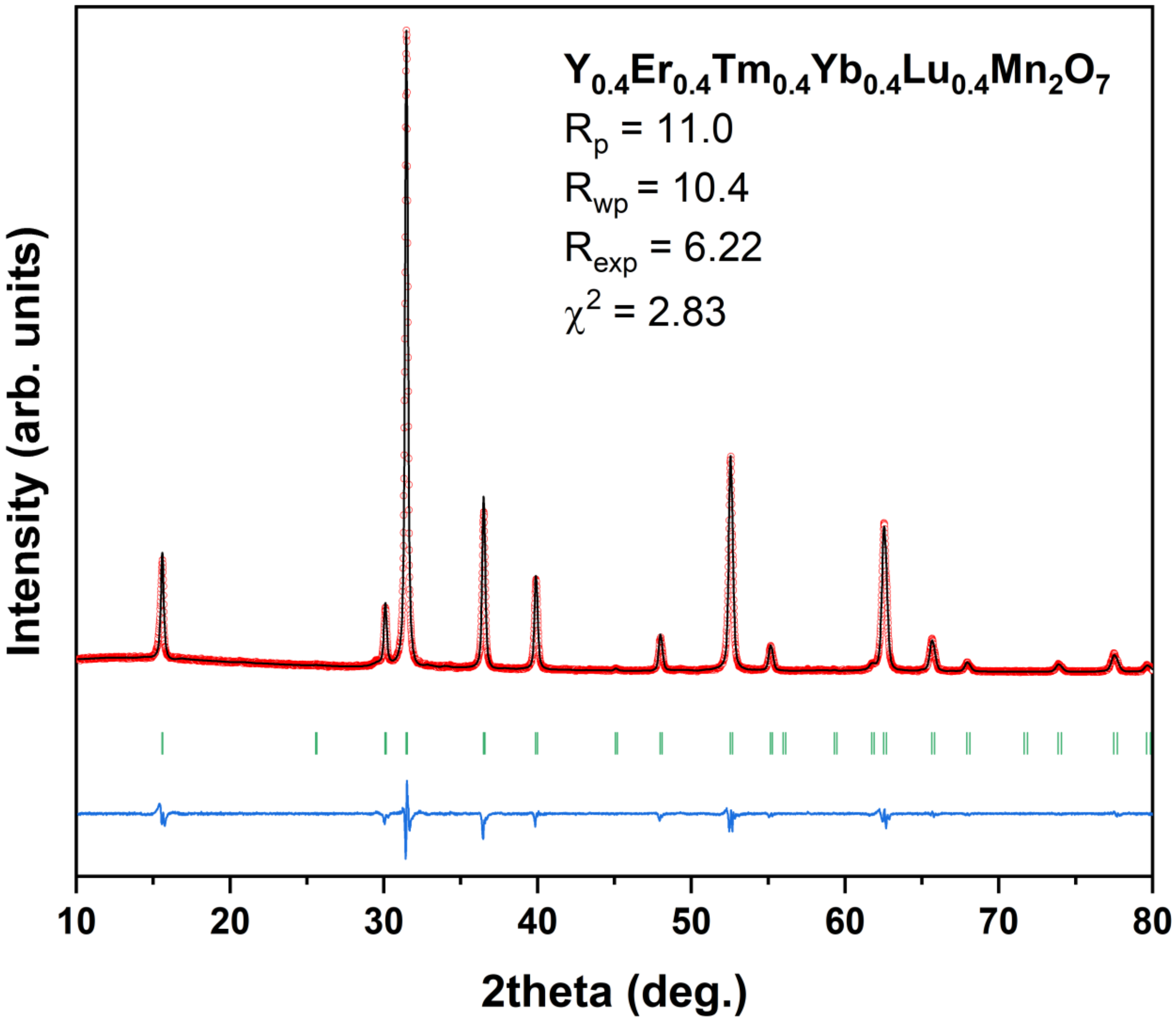


Figure S26. Rietveld refinement data for $Y_{0.4}Er_{0.4}Tm_{0.4}Yb_{0.4}Lu_{0.4}Mn_2O_7$. The red circle symbols and the black solid line represent the experimental and calculated intensities, respectively, and the blue line below is the difference between them. The green tick marks indicate the positions of the Bragg peaks.

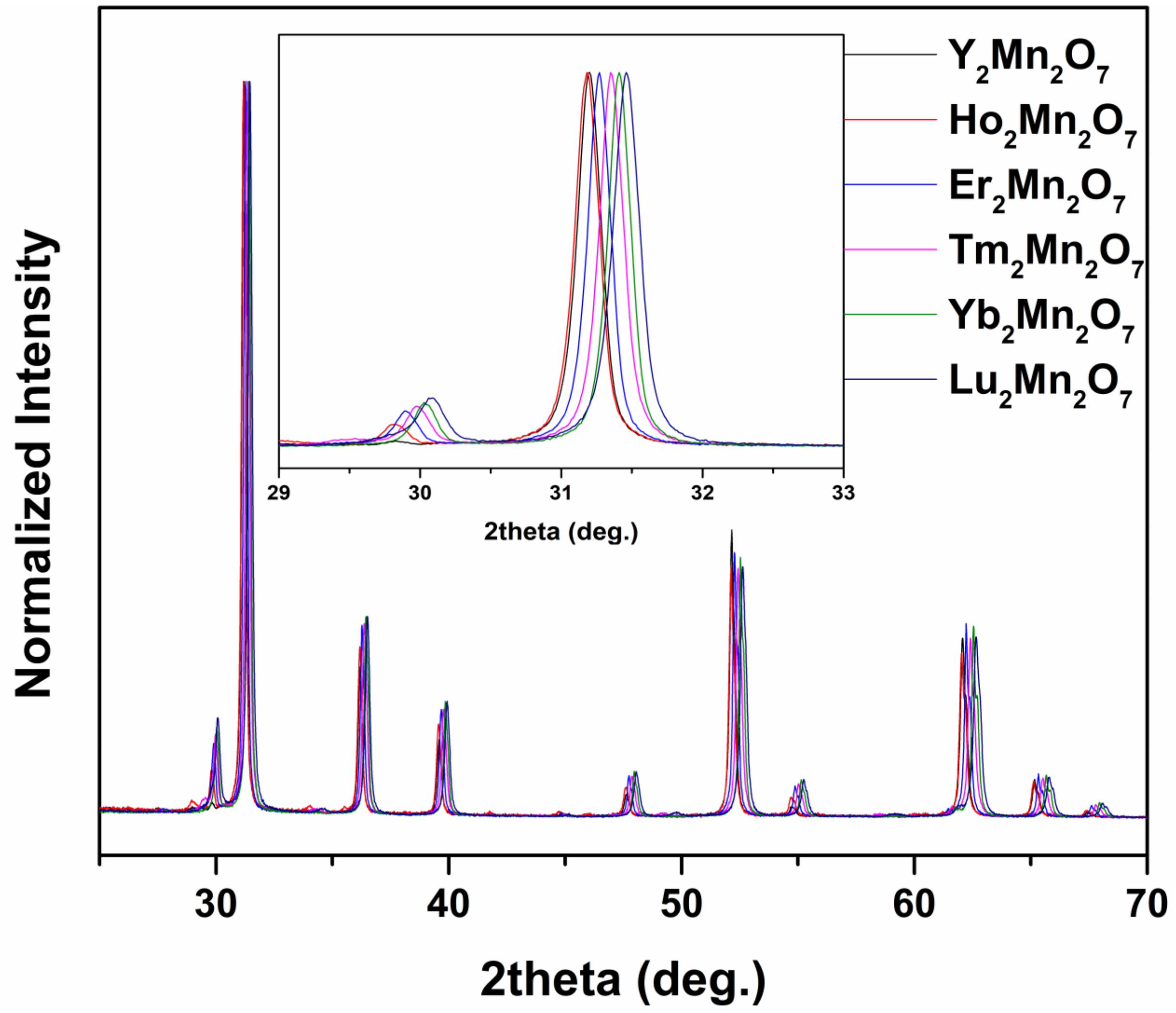


Figure S27. XRD patterns of pyrochlores demonstrating a systematic peak shift.

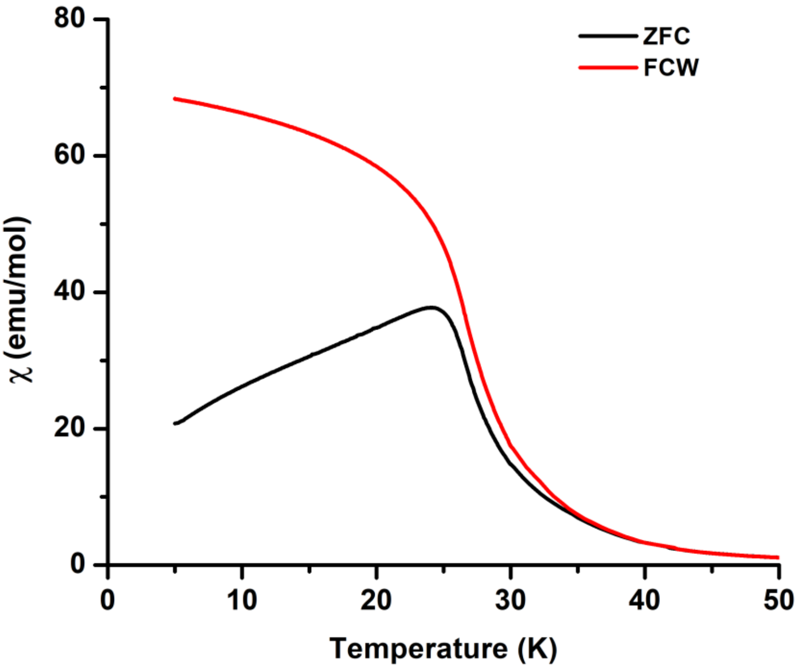


Figure S28. Magnetic susceptibility vs T of $Y_{0.4}Er_{0.4}Tm_{0.4}Yb_{0.4}Lu_{0.4}Mn_2O_7$ at 50 Oe for ZFC and FC data.

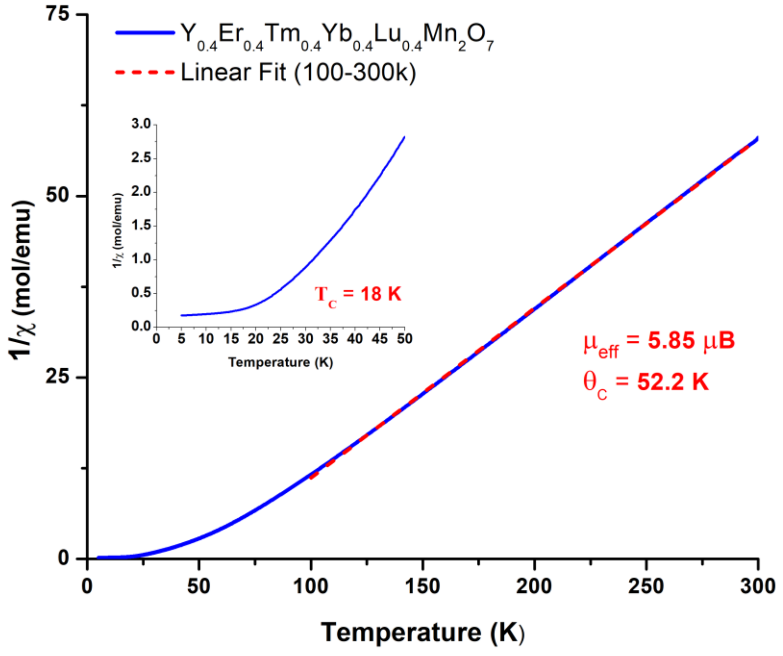


Figure S29. Inverse magnetic susceptibility vs T of $Y_{0.4}Er_{0.4}Tm_{0.4}Yb_{0.4}Lu_{0.4}Mn_2O_7$ at 5000 Oe with Curie-Weiss analysis and linear fit from 100 to 300 K. Inset: Zoomed-in lower T data to show apparent $T_c$.
